# Coordinate Gene Regulation during Hematopoiesis Is Related to Genomic Organization

**DOI:** 10.1371/journal.pbio.0050309

**Published:** 2007-11-20

**Authors:** Steven T Kosak, David Scalzo, Sam V Alworth, Fusheng Li, Stephanie Palmer, Tariq Enver, James S. J Lee, Mark Groudine

**Affiliations:** 1 Division of Basic Sciences, Fred Hutchinson Cancer Research Center, Seattle, Washington, United States of America; 2 SVision, Bellevue, Washington, United States of America; 3 MRC Molecular Haematology Unit, Oxford University, United Kingdom; 4 Department of Radiation Oncology, University of Washington School of Medicine, Seattle, Washington, United States of America; National Cancer Institute, United States of America

## Abstract

Gene loci are found in nuclear subcompartments that are related to their expression status. For instance, silent genes are often localized to heterochromatin and the nuclear periphery, whereas active genes tend to be found in the nuclear center. Evidence also suggests that chromosomes may be specifically positioned within the nucleus; however, the nature of this organization and how it is achieved are not yet fully understood. To examine whether gene regulation is related to a discernible pattern of genomic organization, we analyzed the linear arrangement of co-regulated genes along chromosomes and determined the organization of chromosomes during the differentiation of a hematopoietic progenitor to erythroid and neutrophil cell types. Our analysis reveals that there is a significant tendency for co-regulated genes to be proximal, which is related to the association of homologous chromosomes and the spatial juxtaposition of lineage-specific gene domains. We suggest that proximity in the form of chromosomal gene distribution and homolog association may be the basis for organizing the genome for coordinate gene regulation during cellular differentiation.

## Author Summary

How are genomes—and the chromosomes that comprise them—organized in the eukaryotic nucleus? This long-standing question in cell biology has gained renewed interest due to observations that gene regulation is correlated with the nonrandom distribution of gene loci linearly along chromosomes and spatially within the nucleus. We have used an in vitro model of cellular differentiation to test the hypothesis that there is an inherent organization of the genome related to coordinate gene regulation. Our analysis reveals that during the differentiation of a murine hematopoietic (blood-forming cell) progenitor to derived cell types, co-regulated genes have a marked tendency to be proximal along chromosomes in the form of clusters (of two and three genes) and large-scale domains. Overall gene expression is also spatially proximal, with a pronounced concentration in the nuclear center. The chromosomes themselves parallel this organization of gene activity, with chromosome territories localizing primarily in the interior of the nucleus. Surprisingly, we found that homologous chromosomes have a tendency to be associated, the extent of which is related to the number of co-regulated genes residing on the particular chromosome. Furthermore, individual gene domains display lineage-specific proximity according to their co-regulation. Our study supports the idea that the eukaryotic nucleus is broadly organized—with proximity playing a key role—to facilitate coordinated gene regulation during cellular differentiation.

## Abstract 3

Coordinate gene regulation is required for cellular differentiation. Is the genome nonrandomly organized to accomplish this?

## Introduction

The nucleus appears to be organized according to the many functions it performs [1, 2]. The nucleolus, for example, is a subcompartment that exists as a result of its activities: rDNA transcription and ribosomal biogenesis [1]. Gene loci reflect this functional organization in that their subnuclear localization often correlates with their expression status. Among many examples, it has been demonstrated that: (1) silent loci positioned at the nuclear periphery relocalize to the nuclear center when activated during cellular differentiation (e.g., [3,4]); (2) subsets of expressed genes from a single chromosome territory (CT) colocalize in transcription factories [5]; and (3) the regulation of cell-type–specific genes correlates with their association in the nucleus, despite being found on different chromosomes [6]. In addition, gene loci are often localized relative to their respective CT, with active gene domains looped away from the territory and inactive domains at its surface (e.g., [7,8]). These observations and others have rekindled interest in a long-standing question in the study of nuclear organization: do chromosomes have defined positions within the nucleus?

Structural arrangements of chromosomes, such as the Rabl configuration and the prometaphase rosette, have been known for some time, and there are recent examples of the nonrandom organization of chromosomes [9]. Although it has become clear that nuclear organization is inherently probabilistic, the tendencies for certain chromosomes to be preferentially localized within the nucleus have been demonstrated. For example, analysis of the radial positioning of individual CTs within human nuclei revealed that gene-dense chromosomes have a propensity to be centrally localized, whereas gene-poor chromosomes are more peripheral [10–12]. This phenomenon has also been observed in the nuclei from other primates [13]. An examination of the organization of all chromosomes within individual human nuclei, however, did not reveal a consistent role for gene density in CT localization [14]. Rather, this analysis determined that a chromosome's size (as a function of its overall length) is also related to its radial positioning, with small chromosomes being found more centrally positioned. Similar results were observed in an analysis of mouse nuclei [15]. The varying impact of chromosome density and size may be due to cell-type differences or to the method of analysis (e.g., focusing on a chromosome's center of gravity as opposed to its total area or volume). Nevertheless, a common basis for nonrandom chromosome organization beyond basic chromosome characteristics such as gene density or overall length has yet to be elucidated.

Analysis of genomes from multiple species has revealed that genes have a particular linear arrangement along chromosomes: the co-regulated genes of transcriptomes have a marked tendency to be found grouped (or clustered) according to their shared expression status [2, 16–18]. Therefore, gene loci not only localize to positions within the nucleus relevant to their expression, but they are also inherently organized nonrandomly along chromosomes. It is unclear what function this clustering of genes plays, although the prevailing model suggests that clusters create expression “hubs” or “neighborhoods” in which the linearly proximal genes alter the dynamics of regulatory protein (transcription factor) binding by increasing the relative abundance of binding sites [19]. Given that hundreds of genes are regulated during cellular differentiation, the localization of individual gene clusters may be reflected in the organization of chromosomes enriched in these co-regulated genes [20]. Specifically, chromosomes may be organized in relation to their total, cell-specific expression profile. This organization may involve nuclear localization of chromosome territories, interchromosomal interactions, or both.

We have used an in vitro model of murine hematopoiesis to test the hypothesis that cellular differentiation is associated with a relationship between the linear arrangement of co-regulated genes and chromosomal organization. FDCPmix cells are nontransformed, multipotential hematopoietic progenitors that can be maintained and differentiated into a number of blood cell lineages—including highly pure populations (∼90%) of erythrocytes and neutrophils—with the appropriate cytokines [21]. To explore the possibility of a link between gene expression and genomic organization, we determined the linear chromosomal arrangement of co-regulated genes and the organization of chromosomes for the three cell types. Our results demonstrate a relationship between the chromosomal distribution of co-regulated genes and the propensity for homologous chromosomes and co-regulated gene domains to be proximal. We suggest that the spatial proximity of genes along chromosomes and the association of homologous chromosomes help ensure the coordinate regulation of genes during cellular differentiation.

## Results

### Genes Co-Regulated during Hematopoiesis Are Linearly Proximal along Chromosomes

**Figure 1:**
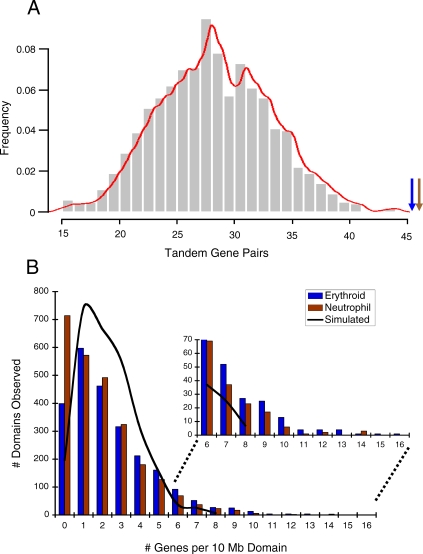
Co-Regulated Erythroid and Neutrophil Genes Are Proximal along Chromosomes (A) The bar graph represents the number of tandem gene pairs from the simulation (Materials and Methods), with a red trend line indicating a Gaussian distribution. The blue arrow indicates the 48 gene pairs found in the erythroid co-regulated gene set, and the brown arrow indicates the 49 neutrophil tandems (χ^2^, *p* < 0.0001). The gene sets are also enriched for triplets (χ^2^, *p* < 0.02). (B) We compared the distribution of the lineage-co-regulated genes to the simulated dataset by sliding-window analysis, with a 10-Mbp window moved in 1-Mbp steps through the genome. A proportions test indicates a significant difference between the lineage and the microarray gene distributions (*p* < 1.2 × 10^−05^), but not between the simulated and microarray (*p* < 0.22). The simulated dataset, represented as a black line, yields a Gaussian distribution. The inset reveals that the lineages have more gene-dense domains than the simulated dataset.

Using data from a microarray analysis of gene expression along a time course of differentiation (Figure S1A) [22], we analyzed the linear chromosomal distribution of co-regulated genes from the FDCPmix cells (hereafter referred to as progenitors) and the derived erythroid and neutrophil cell types. By using Affymetrix databases, the National Center for Biotechnology Information (NCBI) mouse genome alignment (32v1), and BLAT (BLAST-like alignment tool) run locally, we assigned 93% of the ∼12,000 genes represented on the MG-U74Av2 chip to their linear chromosomal positions. We next assigned the erythroid and neutrophil co-regulated genes (or gene sets) to their linear positions. To determine whether the clustering of co-regulated genes is also true in a mammalian differentiation model, we compared the linear distribution of the 594 erythroid and 539 neutrophil genes with lineage-specific expression patterns (Table 1 and Figure S1B) to a simulated gene set, created by the random positioning of “genes” in the ∼11,000 assigned microarray positions and iterated 1,000 times (Materials and Methods). Performing a χ^2^ analysis with the simulated gene set and those of the lineages, we observed that the frequency of co-regulated genes grouped without an interceding unregulated microarray gene is significantly larger in each lineage than predicted by the simulation (*p* < 0.0001) (Figure 1A). We found that 18% (106/594) of erythroid and 20% (106/539) of neutrophil genes are found in clusters of two and—to a lesser degree—three. Interestingly, the examples from other species of co-regulated gene clustering found similar percentages [2]. We verified that the clusters were not due to duplications by removing all redundant GenBank accessions and eliminating any microarray sequences which overlapped with more than one gene (using BLAT) or had any shared sequence identity. These results show that the spatial proximity of co-regulated genes extends to vertebrates and the differentiation of multipotent progenitors extends to derived cell types. Furthermore, the majority (77%) of the co-regulated genes in the erythroid lineage are down-regulated (Figure S1B), demonstrating that in addition to gene activation, clustering may play a role in gene silencing.

**Table 1:**
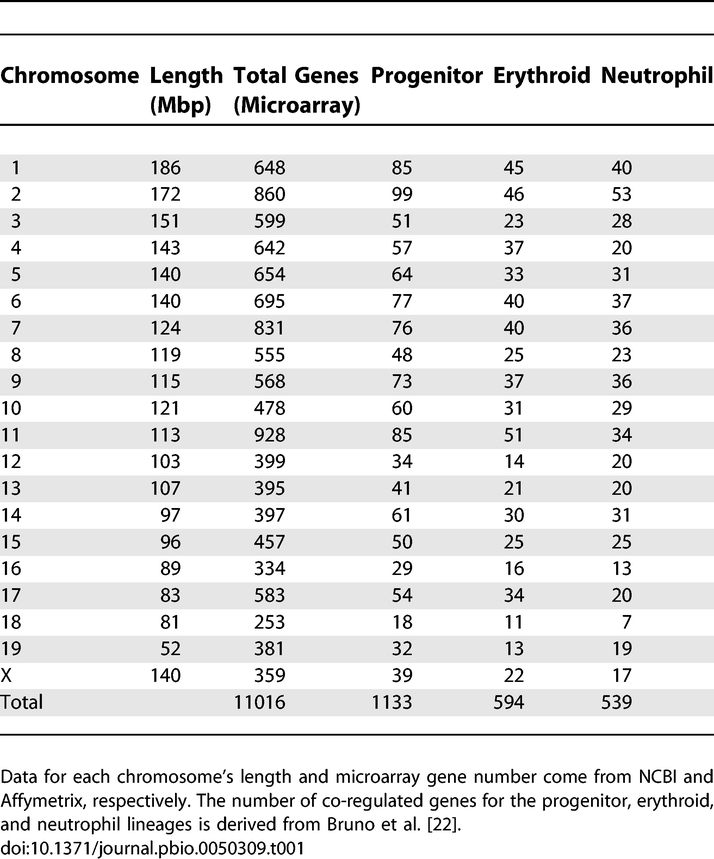
Chromosome Characteristics and Co-Regulated Gene Distribution

Considering the expanse of the entire genome, gene tandems and triplets represent relatively small stretches of DNA. To examine the linear organization of genes beyond clusters, we performed a sliding-window analysis to compare the entire erythroid, neutrophil, and simulated genomic gene distributions to that of the microarray (Figure 1B). Our sliding-window approach—in which a 10–megabase pair (Mbp) window is moved in 1-Mbp increments—helps overcome the relative infrequency of lineage-specific/simulated genes compared with the gene number represented on the microarray chip (∼600 versus ∼11,000). Furthermore, the 10-Mbp window provides a biologically relevant frame, because comparing the murine and human genomes revealed the two share syntenic domains of ∼10–15 Mbp, which suggests a functional constraint on gene domain size [23]. In a proportions test, the distribution of erythroid and neutrophil co-regulated gene sets differed significantly from the microarray (*p* < 1.2 × 10^−35^), whereas the simulated gene set did not (*p* < 0.22) (Figure 1B). The tendency of the lineage gene sets toward gene-dense domains drives their difference with the simulation (Figure 1B, insert). Therefore, beyond tandems and triplets, there is an inherent propensity for the lineage-co-regulated genes to exhibit genomic proximity in domains.

**Figure 2:**
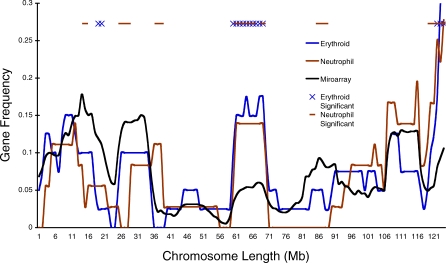
The Lineage-Specific Co-Regulated Genes Have Unique Chromosomal Distributions The erythroid, neutrophil, and microarray sliding-window gene distributions (Figure 1B) were plotted along all of the chromosomes (Figure S2). The sliding-window analysis is depicted along Chromosome 7, demonstrating the linear gene frequency of the microarray and co-regulated genes from the lineages (genes in the window are represented as a ratio of total genes of the microarray or lineage, respectively). An exact binomial test reveals regions of the chromosome that are either significantly enriched or depleted for lineage genes as compared to the microarray, X for erythroid and — for neutrophil. In total, there are 31 erythroid and 37 neutrophil domains across the genome (with at least one domain on most chromosomes) that are enriched for lineage-specific genes (Figure S2).

To visualize the gene distributions, we plotted the erythroid, neutrophil, and microarray sliding-window data along the chromosomes (Figure 2; for all chromosomes, see Figure S2). As the Chromosome 7 example indicates, the mouse chromosomes display regions that are gene dense and gene poor both for the microarray and the co-regulated gene sets from the two lineages (Figure 2). This chromosome structure was first characterized in the analysis of the human genome, wherein gene-dense domains were shown to also be regions of increased gene activity (RIDGEs), whereas the gene-poor regions (or valleys) have little gene activity [24]. In our comparison, there are a number of lineage-specific regions with significantly greater and fewer co-regulated genes than expected by the microarray (Figure S2). For example, the region between ∼ 61 and 71 Mbp is enriched in both lineages for genes that share lineage-specific regulation—either silencing (erythroid) or activation (neutrophil) (Figure S3). Despite these significant differences, however, the gene distributions of the lineages generally follow that of the overall microarray profile, because the significant difference in gene distributions exhibited in the proportions test described above imply a greater tendency for co-regulated gene density, not that the domains are necessarily different from the total gene distribution (depicted on the microarray). Regardless, these data indicate that large-scale, nonrandom gene domains characterize the linear structure of murine chromosomes as well as the distribution of co-regulated genes during differentiation.

### Coordinate Gene Expression and CTs Are Enriched in the Nuclear Interior

**Figure 3:**
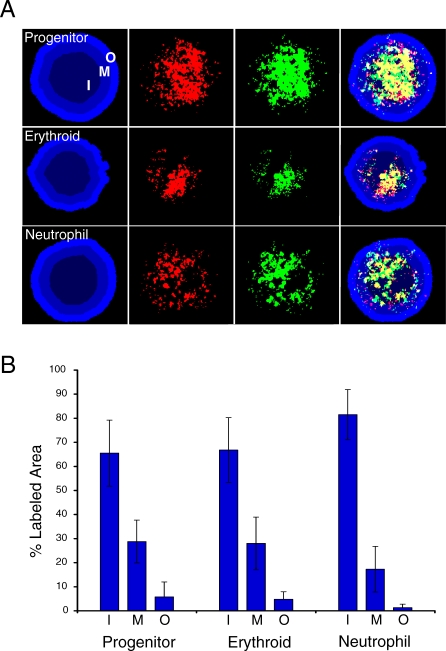
Gene Expression Is Concentrated in the Nuclear Center We analyzed the organization of total gene activity in the progenitor, erythroid, and neutrophil cell types through a modified FISH approach (Materials and Methods). (A) Progenitor, erythroid, and neutrophil nuclei hybridized with biotin- (green) and digoxigenin-conjugated (red) probe material generated from cDNA isolated from each cell type. Cohybridizing with differently labeled probes controls for the specificity of detection (i.e., yellow indicates the degree of overlap for the two probes). The nucleus is counterstained with 4-,6-diamidino-2-phenylindole (DAPI), and the nucleus is divided into three areas of equal area, indicated as inner (I), middle (M), and outer (O). An algorithm in SVCell was created to make these shells and determine the percentage of pixels of the probe material that is localized to the three nuclear regions. (B) Bar graph of results expressed as the percentage of total area in each region. At least 30 nuclei were analyzed per lineage; lines indicate standard deviation.

There are many examples of gene loci demonstrating activity-dependent nuclear localization; therefore, we hypothesized that the complement of expressed genes in the three cell types of our differentiation model may exhibit an inherent nuclear localization pattern. To test this idea, we generated probes for fluorescence in situ hybridization (FISH) that detect total gene expression in the progenitor, erythroid, and neutrophil cell types (Figure 3A) (Materials and Methods). We prepared double-stranded DNA from cDNA prepared from each lineage and used it to amplify probe material through a modified protocol for chromatin immunoprecipitation (ChIP) microarray analysis [25], incorporating either biotin- or digoxigenin-conjugated nucleotides. We analyzed the percentage of probe material represented in three concentric nuclear shells of equal area in two-dimensional (2-D) images (Text S1). In all three cell types, the hybridizations revealed the preferential localization of active genes in the inner nuclear shells, with the innermost shell making up the majority of probe signal (Figure 3B). Importantly, the probe materials that were produced with two different conjugated nucleotide tags were concurrently detected in each nucleus to verify the hybridization patterns (Figure 3A). Therefore, beyond the examples of individual loci, lineage-specific gene expression appears to be spatially organized in the nuclear center. These results are in agreement with bromodeoxyuridine (BrdU)-incorporation analysis, which has indicated that early-replicating chromatin (active or euchromatic) is centralized in the nucleus, whereas late-replicating chromatin (silent or heterochromatic) is enriched in the nuclear periphery (e.g., [26]). Furthermore, the concentration of active gene expression may parallel the role of proximity in the linear chromosome organization of co-regulated genes described above.

**Figure 4:**
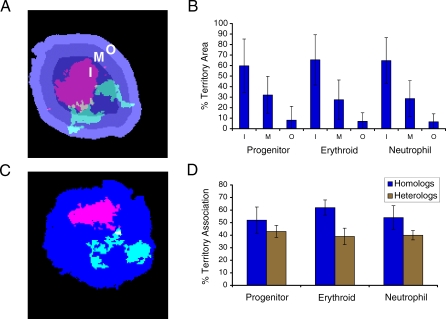
CTs Are Enriched in the Nuclear Center with a Propensity for Homolog Association (A) Erythroid nucleus counterstained with DAPI and CTs 11 (red) and 2 (green) detected by FISH. The nuclear area is divided into three shells—I, M, and O—of equal area as described in Figure 3. (B) Bar graph of resulting means for the analysis of CT positioning for Chromosomes 2, 3, 4, 5, 6, 7, 11, 12, 14, 17, and 19 in the three lineages (for individual chromosome data, see Figure S4). (C) Erythroid nucleus counterstained with DAPI and CTs 7 (red) and 19 (green) detected by FISH. Homologous and heterologous associations were measured by intensity thresholding to remove background and to create a mask of the CT area. Associations were measured only for merged masks (indicated in yellow for the 7 and 19 association). (D) Bar graph of results for the analysis of CT association for chromosomes indicated above (homologous association) and pair-wise analysis of chromosome pairs 2–11, 3–6, 4–5, 7–19, 12–14, 17–19 (heterologous association) as a mean for all analyzed chromosome relationships in the three lineages (for individual chromosome data, see Figure S6A and S6B). At least 30 nuclei were analyzed from each lineage; lines indicate SD.

As indicated in the introduction, previous studies of human chromosomes have alternately found density or length playing a role in their radial localization within the nucleus. In contrast, our analysis above indicates a preference for expressed genes to localize to the nuclear center. Although mouse chromosomes are more uniform than their human counterparts, they still vary widely in their degree of density and length (Table 1). To evaluate the relative importance of these characteristics, we determined the localization of CTs in our differentiation model by performing 2-D FISH on the three lineages with a representative battery of whole-chromosome probes (or paints) (2, 3, 4, 5, 6, 7, 11, 12, 14, 17, and 19, which include short, long, and gene-dense/-poor chromosomes) (Table 1). As in the above analysis of gene expression, we measured the percentage of CTs in three concentric nuclear shells of equal area (Figure 4A). Unexpectedly, all chromosomes showed a significant enrichment in the central portion of the nucleus when compared with the middle or outer shells, regardless of cell type or chromosome size/density (analysis of variance (ANOVA), *p* < 0.0001) (Figure 4B; for individual chromosomes, see Figure S4). The inner and middle regions together compose the vast majority of each CT area. We corroborated our results in nuclei prepared to preserve their 3-D structure (Figure S5A), and we found no significant difference between the 2-D analysis and the CT localization in concentric shells of the six faces of the nuclei sphere (Figure S5B–S5D). That the outer region demonstrates that the lowest percentage of CT area may be linked to the observation that the nuclear periphery is enriched in heterochromatin [27], which is not detected by chromosome paints. Moreover, the central localization of chromosomes may be related to this region's described transcriptional permissiveness (Figure 3A) [2]. The presence of nucleolar organizing regions does not account for this localization, because in the mouse, rDNA is found primarily on the smallest chromosomes and we do not see a relationship with size [27]. Although all the chromosomes we analyzed have their area enriched in the nuclear center, three of the five densest mouse chromosomes (2, 7, and 17) demonstrate an even greater concentration in the inner region (Figure S4). Since these chromosomes are gene dense, they also have a proportionately large number of the lineage-specific genes (Table 1 and Dataset S1). Therefore, the demonstrated tendency of gene-dense human chromosomes to be localized in the nuclear center may be due to their having the greatest number of active genes in any particular cell type. Further research will be necessary to understand fully the radial organization of CTs and the function it may play in coordinate gene regulation.

### Homologous Chromosomes Demonstrate a Tendency to Associate

When analyzing CT radial distribution, we observed the tendency for homologous chromosomes to be in proximity of each other (Figure 4A). Therefore, using the images from the radial analysis, we measured the interaction of homologs through intensity thresholding, with CTs being scored as associated only if the above background pixels were unambiguously connected (Figure 4C). This stringent criterion reveals that chromosomes show a high frequency of homologous interaction in the interphase nucleus. An average of 50% of nuclei in each lineage display homologous chromosome association, with variations among individual chromosomes (Figure 4D; for individual chromosomes, see Figure S6A). Furthermore, the results are not due to the 2-D approach, because we also analyzed homolog association through the depth of nuclei prepared to preserve their 3-D structure and found no significant difference in the results (Figure S7A and S7B). The association for pairs of heterologous chromosomes was also measured, demonstrating a high degree of interaction (on average ∼40%) (Figure 4D; for each pair, see Figure S6B). However, because there is twice the possibility of interaction between two heterologous chromosome pairs than a single homologous pair, these data support the prevalence of homologous chromosome association. We suggest that homolog proximity may be related to the propensity for CTs to be localized to the nuclear center and to the chromosomal distribution of co-regulated genes.

To determine whether the association of homologs is correlated to their number of co-regulated genes, chromosome gene density, or chromosome size (length), we performed a Kruskal-Wallis (K-W) test comparing these chromosomal attributes (Figure 4D and Table 1). A multivariate statistical analysis, the K-W test is a one-way ANOVA by ranks, in which each dataset is ranked in a column—according to chromosome number—and then statistically analyzed in rows across values (or the conditions of co-regulated gene number, proximity, and length). For the three lineages, there is a striking pattern of significance in that the proximity of homologous chromosomes is related only to the chromosomal distribution of co-regulated genes (Table 2; for the rankings, see Figure S8). The erythroid cells, e.g., reveal that the ranking of chromosomes for homologous proximity does not significantly differ from the ranking of chromosomes for their distribution of co-regulated genes, yet it does for the basic characteristics of size and density (Table 1). Therefore, the distribution of co-regulated genes appears uniquely related to the proximity of homologous chromosomes, underscoring the importance of proximity in coordinate gene regulation.

**Table 2:**
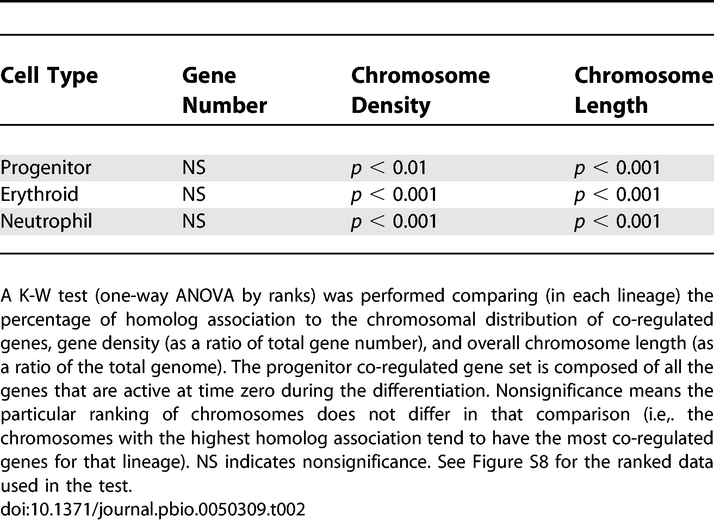
The Association of Homologs and Chromosomal Co-Regulated Gene Distribution Are Related

### Gene Clusters Are Spatially Proximal in the Nucleus According to Their Co-Regulation

Coordinate gene expression has been thought of as a type of network, because it is composed of genes (or nodes) that are related (linked) in terms of their co-regulation and shared function, such as in the differentiation of a given cell type. In real-world networks—shown to be prevalent in biological systems—a diminishing number of nodes with an increasingly greater number of links create a hub organization [28]. The lineage-specific erythroid and neutrophil linear chromosomal gene distributions exhibit this characteristic, with their sliding window data demonstrating a negative power-law degree-distribution (erythroid P(*k*) = *k* ^–2.6^, neutrophil P(*k*) = *k* ^–2.5^). This behavior provides a basis for modeling gene regulation during differentiation, emphasizing the importance of linear proximity and suggesting that spatial proximity of gene domains may also play an important role in coordinate gene regulation. Specifically, the prevalence of homolog association may be related to the proximity of the co-regulated gene clusters within the nucleus.

**Figure 5:**
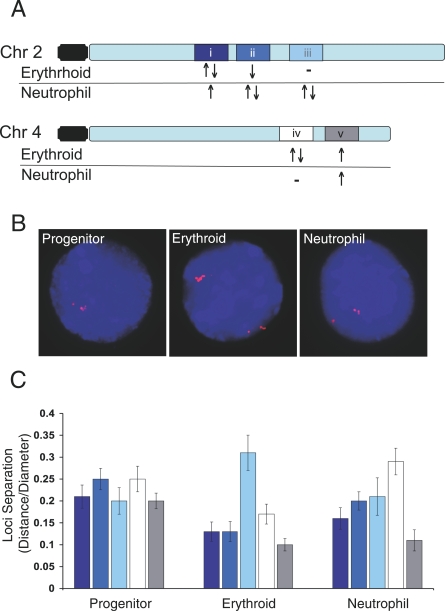
Gene Domains Exhibit Differential Nuclear Proximity According to Their Co-Regulation (A) Five chromosome domains (i–v) on two chromosomes were identified with genes co-regulated during differentiation of the progenitors to erythroid and neutrophil lineages (Dataset S1). The domains are composed of genes with shared activation (upward-pointing arrow), silencing (downward-pointing arrow), mixed (upward- followed by downward-pointing arrows), or no co-regulated genes (minus sign). They range from ∼13 to 2 Mbp and are represented by FISH probes generated with multiple bacterial artificial chromosomes (BACs) for each domain. (B) FISH images of nuclei from the three lineages (counterstained with DAPI) hybridized with six BAC probes (red) to domain iii (∼6 Mbp). (C) Bar graph of results for each probe set (i–v) hybridized to nuclei from each lineage. Masks of the FISH signals were generated and the nearest distance between the masks was measured. Distances are expressed as a ratio of the nuclear diameter. At least 30 nuclei from each lineage for each probe set were analyzed; lines represent standard error of the mean.

To test this hypothesis, we arbitrarily identified five gene domains with unique coordinate gene expression in the erythroid and neutrophil cell types on two chromosomes of relatively equal length (2 and 4) (Figure 5A). We analyzed the spatial proximity of the homologous domains—determined as a ratio of distance between domains to nuclear diameter—in the progenitor, erythroid, and neutrophil lineages using 2-D FISH (Figure 5B; verified in 3-D in unpublished data). The co-regulated genes in these domains are all active in the progenitors. Consistent with this shared expression status, the domains do not demonstrate significant differences in their separation in the progenitor nuclei (ANOVA *p* = 0.49 )(Figure 5C). Importantly, however, the spatial proximity of the domains in the erythroid and neutrophil nuclei do differ significantly (ANOVA, *p* < 0.001 and *p* < 0.01, respectively) (Figure 5C). In both lineages, the degree of domain proximity varies according to its overall activity status. For example, the domains without any regulated genes demonstrate the greatest separation, whereas the most active domains are the closest. Analyzing the data for the degree of direct loci colocalization supports the overall behavior of the domains (Dataset S1). Therefore, these results support the hypothesis that the spatial proximity of lineage-specific gene domains may further facilitate the co-regulation of genes colinear along chromosomes.

### Total Chromosome Analysis Supports the Prevalence of Homolog Proximity

**Figure 6:**
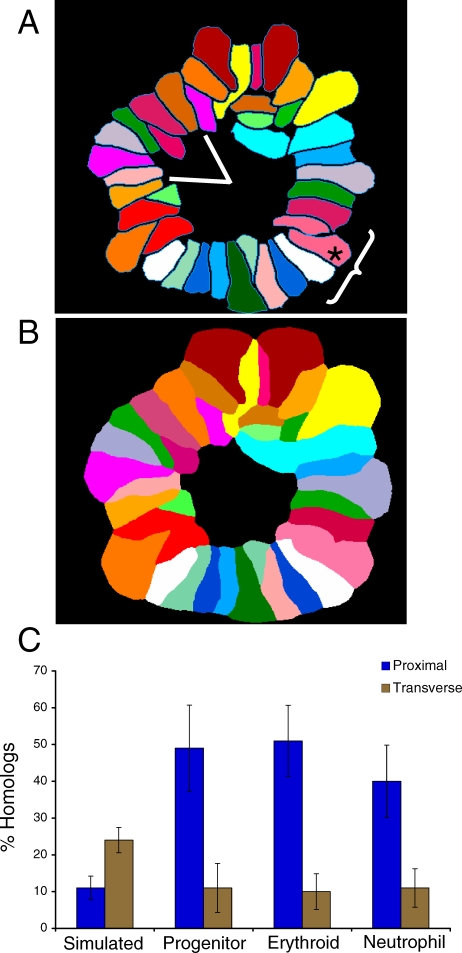
Total Chromosome Analysis Supports the Prevalence of Homolog Proximity We studied chromosome organization by simultaneously detecting all of the chromosomes in lineage-specific rosettes using SKY. (A) We performed two types of analyses on at least 30 rosettes from each cell type and a simulated rosette dataset (Materials and Methods): (1) homologous sister chromatid pairs (homologous chromosomes) were assayed for proximity by determining the frequency of their being within two chromosomes of each other (asterisks indicates the assayed chromosome, the bracket identifies the region of proximity as three chromosomes on either side); (2) homologous chromosomes were scored for being transverse by determining their frequency of being across the centre of a rosette in a 60° angle window (encompassing ∼8 chromosomes). Chromosomes X and Y are not considered homologous and are not a part of this analysis. (B) Illustration of the distance constrained zone-of-influence (ZOI) operation performed by SVCell. The proximity of CTs is calculated automatically by performing a ZOI-based region partition around each CT. The ZOI operation creates an unambiguous representation across which adjacency transitions can be determined. If at least one pixel of two chromosomes' partitioned regions touch, then they are considered proximal. (C) Spatial pattern rules were created in SVCell and used to measure proximal and transverse associations for all chromosomes in each of the rosettes for the three lineages and the simulated dataset. The bar graph depicts these results as a mean of the chromosomal data for each lineage (for individual chromosome data, see Figure S9A and S9B); lines indicate standard deviation.

Given the complexity of chromosome distribution in the interphase nucleus, prior attempts to determine the simultaneous organization of all chromosomes have relied on center-of-gravity measurements [14]. However, this type of analysis does not take into account the contours of a CT, which are relevant in discerning chromosome associations. Therefore, we developed a strategy to analyze the simultaneous relationship of all chromosomes in prometaphase rosettes, when a cell's complement of chromosomes come together to form a circle with their centromeres (Figure 6A). We used spectral karyotyping (SKY) [29]—developed for the clinical detection of chromosomal abnormalities—and implemented a method to perform pattern recognition on SKY rosettes in SVCell software (SVision, Bellevue, WA, United States). Our approach executes distance-constrained, zone-of-interest (ZOI) region partition on the SKY image (Figure 6B), from which chromosome proximity can be automatically determined for all chromosome associations at a resolution of one pixel (for a complete description of the software, see Text S1). Rosettes have long been used to study chromosome organization (e.g., (14,30]), although it remains controversial whether chromosome relationships are maintained through mitosis [31–33]. However, regardless of whether organization is maintained, we examined rosettes to determine if the tendency for homolog proximity is observed under conditions that permit analysis of all chromosomes at the same time.

Since the mouse genome is composed of two complements of 19 autosomes and two sex chromosomes, the likelihood that a chromosome associates with any other in the rosette is at least 2/39 (or 5.1%, associations can occur on either side of the chromosome) (Figure 6A). To examine a region of chromosome association, we defined proximity as being no more than two chromosomes apart along the contours of the territories (Figure 6A). Using this criterion, we compared the association of every chromosome to all others in simulated (Materials and Methods) and lineage rosette datasets. In support of our findings from individual chromosome analysis (Figure 4C and 4D), we observed a high frequency of proximity for homologous chromosomes in the three cell types: on average, homologs associated in 48%, 51%, and 40% of progenitor, erythroid, and neutrophil rosettes, respectively (Figure 6C; for individual chromosome data, see Figure S9A). In comparison, only 11% of homologs associated in the simulated rosettes (ANOVA, *p* ≤ 3.8 × 10^−11^), which reflects the random expectation for our designation of proximity (6/39 or 15.4%) (Figure 6C). Importantly, a significant difference is maintained whether proximity is defined as chromosomes being directly adjacent or one chromosome removed (Dataset S1). Homologous chromosomes vary in their degree of proximity among the three cell types, although there is no size-dependent trend (Figure S9A). An earlier study of rosettes, using individual chromosome paints, had found that homologs tend to be located across the center of the rosette (or transversely related) [34]. To exclude this possibility and to verify our observation of proximal association, we measured the number of homologs separated by at least 2.618 radians (or an angle of 60°) across the rosette center from the chromosome analyzed (Figure 6A). The simulated rosette set closely follows the prediction of 21% for random association (a 60° angle includes ∼8 chromosomes, 8/39, 24%) (Figure 6C). Homologous chromosomes in the lineages, however, show a significantly lower degree of transverse separation than the simulated dataset does (ANOVA, *p* < 4.7 × 10^−8^), corroborating our determination of proximity (Figure 6C; for individual chromosome data, see Figure S9A). A K-W test comparing proximal homologs, transverse homologs, chromosome gene density, and chromosome size support the conclusion that co-regulated gene distribution is uniquely related to the association of homologs (Table S1).

## Discussion

By combining analyses of gene expression patterns and chromosome localization, we have tested the hypothesis that coordinate gene regulation during cellular differentiation is related to a specific organization of the genome. Like examples from other organisms, we found that genes co-regulated during murine hematopoiesis are significantly colinear, forming gene clusters along chromosomes (Figure 1). Furthermore, we determined that clustering is not limited to gene activation, because the erythroid lineage is characterized by gene silencing and displays a similar degree of clustering as neutrophils do. Beyond the adjacency of individual genes, we found a wide-spread tendency for the co-regulated gene sets to reside nonrandomly in large gene domains (Figure 2). Reasoning that the examples of individual loci exhibiting lineage-specific nuclear positions may be broadly reflected in active gene localization during cellular differentiation, we developed an approach to determine the nuclear distribution of a cell type's complement of expressed genes. Our results revealed that the nuclear interior is not only transcriptionally permissive, but the preferred region for coordinate gene expression (Figure 3). This pattern of localization is mirrored in the chromosomes themselves, with CTs being enriched in the nuclear center (Figure 4B). Interestingly, this preference for central positioning of CTs is coupled with a propensity for homologous chromosomes to interact (Figure 4D). The degree of homolog association is related to the chromosomal distribution of co-regulated genes (Table 2), and representative gene domains analyzed by FISH exhibit greater spatial proximity in the nucleus according to their lineage-specific expression patterns (Figure 5). Finally, by using a novel means of analyzing the simultaneous organization of chromosomes, we corroborated the tendency for homologs to be proximal (Figure 6). Therefore, despite its complexity and probabilistic nature, the nucleus appears to be nonrandomly organized for coordinate gene regulation.

Our analysis suggests that the co-regulated gene distributions of the erythroid and neutrophil lineages can be described as scale-free networks. Beyond their temporal regulation and chromosomal distributions, the lineage-specific gene domains also demonstrate physical proximity within the nucleus, underscoring their regulatory linkage. An important feature of networks—in particular those that are scale-free—is their tendency for self-organization. We suggest that transcriptional regulation during cellular differentiation is related to the nucleus self-organizing according to the principle of proximity [1]. Extending studies demonstrating that X inactivation is related to the physical interaction of the X chromosomes [35,36], we argue that the association of homologous chromosomes is widespread and correlated with the proximity of similarly regulated gene domains during cellular differentiation. Therefore, homolog association may facilitate the formation of expression hubs containing the alleles of co-regulated gene domains [2]. Hematopoietic progenitors have been shown to undergo “lineage priming,” a low-level promiscuous expression of genes expressed in differentiated cell types [37,38]. The role that lineage priming may play in gene expression during differentiation remains to be determined. However, given the high diffusion rate of regulatory [39] and structural [40] nuclear proteins, it is attractive to consider that spatial proximity would alter the off-rate for DNA-binding proteins, creating localized protein concentrations—such as in the nuclear center—to ensure the co-regulation of relevant gene sets. Therefore, beyond its central role in the homologous recombination that helps fuel variation and natural selection, diploidy may also be involved in facilitating the co-regulation of entire gene sets during cellular differentiation. Whether allelic proximity is a requirement for or a result of transcriptional regulation and the mechanism(s) underlying the association of homologs remain to be established.

## Materials and Methods

### Cell culture.

For the culture and analysis of FDCPmix cells, the progenitors were routinely cultured in Iscove's Modified Dulbecco's (GIBCO) medium supplemented with 20% (vol/vol) horse serum (GIBCO) and 10 ng/ml recombinant murine IL-3 (R&D Systems). Differentiation of the progenitors was performed as previously described [21]. The growth factor concentrations used were as follows: erythroid: Epo (5 U/ml; Amgen), hemin (0.2 mM; Sigma), and rmIL-3 (0.05 ng/ml; R&D); neutrophil: G-CSF (rmG-CSF; 50ng/ml; R&D) and SCF (rmSCF; 100 ng/ml; R&D). Cells were stained with benzidine and cytospins stained with May-Grünwald-Giemsa to verify cellular morphology. In addition, cell lineages were verified on a FACS-Vantage (Becton Dickinson) after staining for cell surface markers, with fluorescein-conjugated mouse monoclonal antibodies directed against Ter-119, Gr-1, or Mac-1 (Pharmingen).

### Bioinformatics analysis.

Combining information from Affymetrix databases and the NCBI mouse genome alignment (32v1) with BLAT run locally, we assigned 93% of the ∼11,000 genes (11,160) represented on the MG-U74Av2 chip to their linear chromosomal positions. Checking for GenBank duplicate entries, we also assigned lineage (erythroid or neutrophil) and differential expression classes (I–IV, zero indicates no change) to these positions (Dataset S1, ComboDatabase sheet). The simulated dataset was created by randomly positioning 650 “genes” in the ∼11,000 microarray positions, iterated 1,000 times. The number 650 was originally chosen, because this was the larger gene set size (of the erythroid and neutrophil) before removal of duplicate accession on the microarray chip.

Using data from this combined dataset, we performed statistical analysis using the R statistical environment or the statistical package XLSTAT (Addinsoft). Data processing— i.e., sliding window, data simulation, or position assignment—was performed using a combination of perl scripts and R. Statistical analysis was performed by extracting relevant data from this larger dataset. The K-means, proportions test, and χ^2^ analyses were performed directly. When a test required an intermediate output from the perl scripts, these intermediate files (e.g., sliding-window datasets) are in Dataset S1. Intermediate files were used to demonstrate power law distributions and to perform exact binomial tests (Dataset S1, Chr#_E&N sheets).

Using Prism (Graphpad Software) we performed a K-W test with a Bonferroni post-test on the nonparametric rosette data (Dataset S1, RosetteData sheet) and ANOVA with Bonferroni post-test for the masked territories (Dataset S1, Mask_Regions%Territory sheet), among other analyses.

### FISH analysis.

Progenitor, erythroid, and neutrophil cells were taken from asynchronously growing cultures at day five of the differentiation. Slides were prepared by either briefly treating with a hypotonic solution (KCl, 0.075 M) and then fixing in at least five changes of 3:1 absolute methanol:glacial acetic acid before spreading, or first adhering the suspension cells to chambered slides using poly-L-lysine followed by hypotonic treatment and fixation. 2-D FISH was performed as described [3] with slight modifications. Briefly, slides were prepared for DNA hybridization by treatment with RNase (100 mg/ml), ethanol washes, and subsequent denaturation in 50% Formamide/50% 2xSSC at 75 °C for 2 min.

To analyze genomic gene expression within the nucleus, we first isolated total RNA from the progenitor, erythroid, and neutrophil cells using TRIzol Reagent (Invitrogen). Subsequently, we generated double-stranded (ds) cDNA using SuperScript (Invitrogen) following the manufacturer's protocol. Importantly, the number of cycles for the amplification has to be empirically determined for each sample. We found that 17 cycles provided a defined spread of products of sufficient quantity. Following ds cDNA production, we slightly modified the protocol used for the amplification of probe material in chromatin immunoprecipitation (ChIP)-microarray analysis [25], by using ds cDNA as input and incorporating biotin- and digoxigenin-conjugated nucleotides through random labeling. Whole chromosome probes (or paints) to various chromosomes (2, 3, 4, 5, 6, 7, 11, 12, 14, 17, and 19) were obtained from either Applied Spectral Imaging or Cambio and were hybridized according to manufacturer's specifications.

Images of single or multiple Z sections were captured on an Olympus IX 70 or a Zeiss Axiovert 100 TV microscope equipped with cooled CCD cameras and operated using Deltavision SoftWorx software (Applied Precision). Deconvolution was performed using AutoDeblur, or in some cases, no deconvolution was used on the images (AutoQuant Imaging). Masking occurred using Imaris software (Bitplane). Masks were created by intensity thresholding to remove background and capture the extent of the gene expression probe material or CT area. For each fluorescent channel, these masks were converted to binary masks and then either exported as TIFs for nuclear region analysis using SVCell (SVision) (Text S1) or assayed for homologous and heterologous CT association (identified by unambiguous contiguity of CTs). At least 30 nuclei were assayed for each type of analysis.

For a detailed description of the 3-D FISH protocol, see Text S1. Image stacks of 30–40 Z sections (spaced 0.25 μm apart) were captured on a Zeiss Axiovert 100 TV microscope equipped with a cooled CCD camera and subsequently deconvolved using AutoDeblur. The 3-D images were analyzed as a projection of the six faces of the nucleus (CT localization) or as a volume (CT association) in Imaris.

### Network analysis.

Random real-world networks have been shown to follow a negative power-law degree-distribution, P(*k*) ∼ *k* ^–*g*^, with a *g* (degree exponent) between 2 and 3. We plotted on a log-scale the number of links (*k*), which in our analysis are the 10-Mb domains from the sliding-window analysis, as a log function of the frequency of those domains with a given density. The distributions of the gene sets conform to the expectations of a scale-free network, with degree exponents of 2.6 (erythroid) and 2.5 (neutrophil) (Dataset S1). Analysis of 1- and 5-Mb domains yields similar distributions (unpublished data).

### Assaying chromosome spatial pattern associations in rosettes.

Rosettes were enriched from asynchronous populations by the preparation of slides the day after splitting the cultures. To be analyzed, the rosettes had to exhibit the characteristic circular shape formed by the centromeres without pronounced perturbations; at least 30 rosettes per lineage were analyzed. The simulated rosettes were created from ten rosettes from the various lineages with their karyotype information removed. Using a random number generator (http://www.randomizer.org), we made 100 sets of random numbers (a set consists of two random lists of 1–20), and moving from one chromosome to another imposed the random number as its chromosome karyotype. SKY hybridization and detection were performed according to manufacturer's specifications (Applied Spectral Imaging).

SVCell alpha prototype software (SVision LLC) was used to assay spatial pattern associations between individual chromosomes across all the rosette images from the three lineages and a simulated rosette image set. SVCell is a microscopy image informatics tool that contains fast image recognition algorithms, relational measurements, and supports the creation and review in real time of a large number of spatial patterns that can be derived from these relational measurements. SVCell image recognition and relational measurements automatically normalize the distortion and intersample variations among input images. We created spatial pattern rules in SVCell and used them to interrogate the rosette images for the pattern's frequency in total and across all chromosome interactions. The application is described in detail in Text S1.

## Supporting Information

Dataset S1 Combined Data for Bioinformatics and Image Analysis (2.8 MB XLS)

Figure S1 The Erythroid and Neutrophil Co-Regulated Gene Sets (A) A microarray expression analysis was performed on the FDCPmix (progenitor) hematopoietic differentiation model, along an eight-point time course (0, 4, 8, 16, 24, 48, 72, and 168 h), into two distinct lineages (erythroid and neutrophil) and two mixed lineages (megakaryocyte/erythroid and monocyte/neutrophil) [22]. The expression profile reveals active (red) and inactive (green) genes, grouped according to the results of a K-means analysis. There are four primary expression classes, I and II are up-regulated, while III and IV lead to down-regulation.
(B) The erythroid and neutrophil lineages are not represented equally in the four different types of expression, with the erythroid cells demonstrating an overall pattern of down-regulation (75% III and IV) and the neutrophils that of up-regulation (59% I and II).
(143 KB PPT)

Figure S2 The Lineage-Specific Co-Regulated Genes Have Unique Gene Distributions The erythroid, neutrophil, and microarray sliding-window gene distributions (Figure 1B) were plotted along all the chromosomes.
(251 KB PPT)

Figure S3 Gene Domains Share Expression Profiles The region significantly enriched for both erythroid and neutrophil genes (between 60 and 78 Mbp) on chromosome 7 (Figure 2) is represented as a sliding-window analysis. The 10-Mbp window is moved in 1-Mbp steps, encompassing 20 steps. This depiction shows that these regions have genes primarily of related expression classes (blue = erythroid, brown = neutrophil), and that through an exact binomial test, these regions differ significantly from the expectation from the microarray.
(30 KB PPT)

Figure S4 Chromosome Territories Are Enriched in the Nuclear Center A battery of chromosomes (2, 3, 4, 5, 6, 7, 11, 12, 14, 17, and 19) was measured for their CT radial localization. An algorithm in SVCell was created to make three shells of equal area to determine the percentage of pixels from CTs localized to the inner (I), middle (M), and outer (O) regions of the nucleus. The entire battery of chromosomes was measured in each of the lineages. Statistical comparisons can be found in Dataset S1.
(79 KB PPT)

Figure S5 3-D Analysis of Chromosome Territory Distribution in the Interphase Nucleus (A) 3-D progenitor nucleus with CTs 7 (red) and 19 (green) detected by FISH and counterstained with 4-,6-diamidino-2-phenylindole (DAPI). The six planes of the 3-D projection are depicted and numbered. Concentric nuclear shells of equal area were analyzed for CT distribution in each of the planes. Territory distribution was then expressed as an average of the six planes for each nucleus.
(B–D) Line graphs comparing the distribution of CTs from the 2-D to the 3-D analysis in the progenitor for (B) chromosome 7, (C) chromosome 14, and (D) chromosome 19. ANOVA with Bonferroni correction reveals no significant difference between the 2-D and 3-D data (*p* > 0.05). At least 15 3-D progenitor nuclei were analyzed for each CT.
(192 KB PPT)

Figure S6 Chromosome Territories Are Enriched in the Nuclear Center with a Tendency for Homolog Association The battery of chromosomes from Figure S4 was measured for the association of (A) homologs and (B) heterologs. Associations were measured by intensity thresholding to remove background and for the creation of a mask of the CT area. Associations were measured only for merged masks. The entire battery of chromosomes was measured for the frequency of homologous association and the representative 2–11, 3–6, 4–5, 7–19, 12–14, and 17–19 pairings were measured for heterologous associations. At least 30 nuclei were measured for each analysis.
(192 KB PPT)

Figure S7 3-D Analysis of Chromosome Territory Association in the Interphase Nucleus (A) 2-D projection (from Imaris) of the 3-D progenitor nucleus from Figure S5. Homologous associations were measured by intensity thresholding to remove background and to create a mask of the CT area. Associations were measured for merged masks through the stack of images or a 3-D projection.
(B) Bar graph of instances of homolog association for three CTs (7, 14, and 19) analyzed in both 2-D and 3-D progenitor nuclei. ANOVA with Bonferroni correction reveals no significant difference between the 2-D and 3-D data (*p* > 0.05). At least 15 3-D progenitor nuclei were analyzed for each CT.
(226 KB PPT)

Figure S8 Ranked Data for K-W Test of Homolog Association and Chromosome Characteristics Analysis was performed with Prism (Graphpad Software), from which the ranked data were extracted. Rankings are in ascending order, with 11 being the greatest (there were 11 chromosomes analyzed). Shared and averaged values among the characteristics result from the ranking procedure.
(50 KB PPT)

Figure S9 Total Chromosome Analysis Supports the Prevalence of Homolog Proximity We performed two types of analyses on at least 30 rosettes from each lineage and a simulated dataset. (A) Homologous sister chromatid pairs (homologous chromosomes) were assayed for proximity by determining the frequency of their being within at least two chromosomes of each other. (B) Homologous chromosomes were scored for being transversely related by determining their frequency of being across the center of a rosette in a 60° angle window.
(264 KB PDF)

Table S1 The Association of Homologs and the Chromosomal Distribution of Co-Regulated Genes Are Related A K-W test comparing the proximal homologous chromosome dataset for each lineage to their transverse chromosome datasets, chromosomal distribution of co-regulated genes (for each cell type), chromosome gene density (as a ratio of total gene number), and overall chromosome length (as a ratio of the total genome) was performed. The progenitor co-regulated gene set is composed of all the genes that are active at time zero during the differentiation. Nonsignificance means that the particular ranking of chromosomes does not differ in that comparison (i.e., the most proximal chromosomes tend to have the most co-regulated genes for that lineage). NS indicates nonsignificance.
(24 KB PPT)

Text S1 Detailed Description of Image Analysis Methods (34 KB DOC)

## Abbreviations

ANOVA: analysis of variance
BLAT: BLAST-like alignment tool
CT: chromosome territory
FISH: fluorescence in situ hybridization
K-W test: Kruskal-Wallis test
SKY: spectral karyotype
ZOI: zone of influence
