# Supplementary material for: Coordinate Gene Regulation during Hematopoiesis Is Related to Genomic Organization
Source: PLoS Biol. 2007 Nov 20;5(11):e309. doi: 10.1371/journal.pbio.0050309 (PMC2080650; doi:10.1371/journal.pbio.0050309)
Supplement: Supplementary file 1 [file pbio.0050309.sg002.pdf]

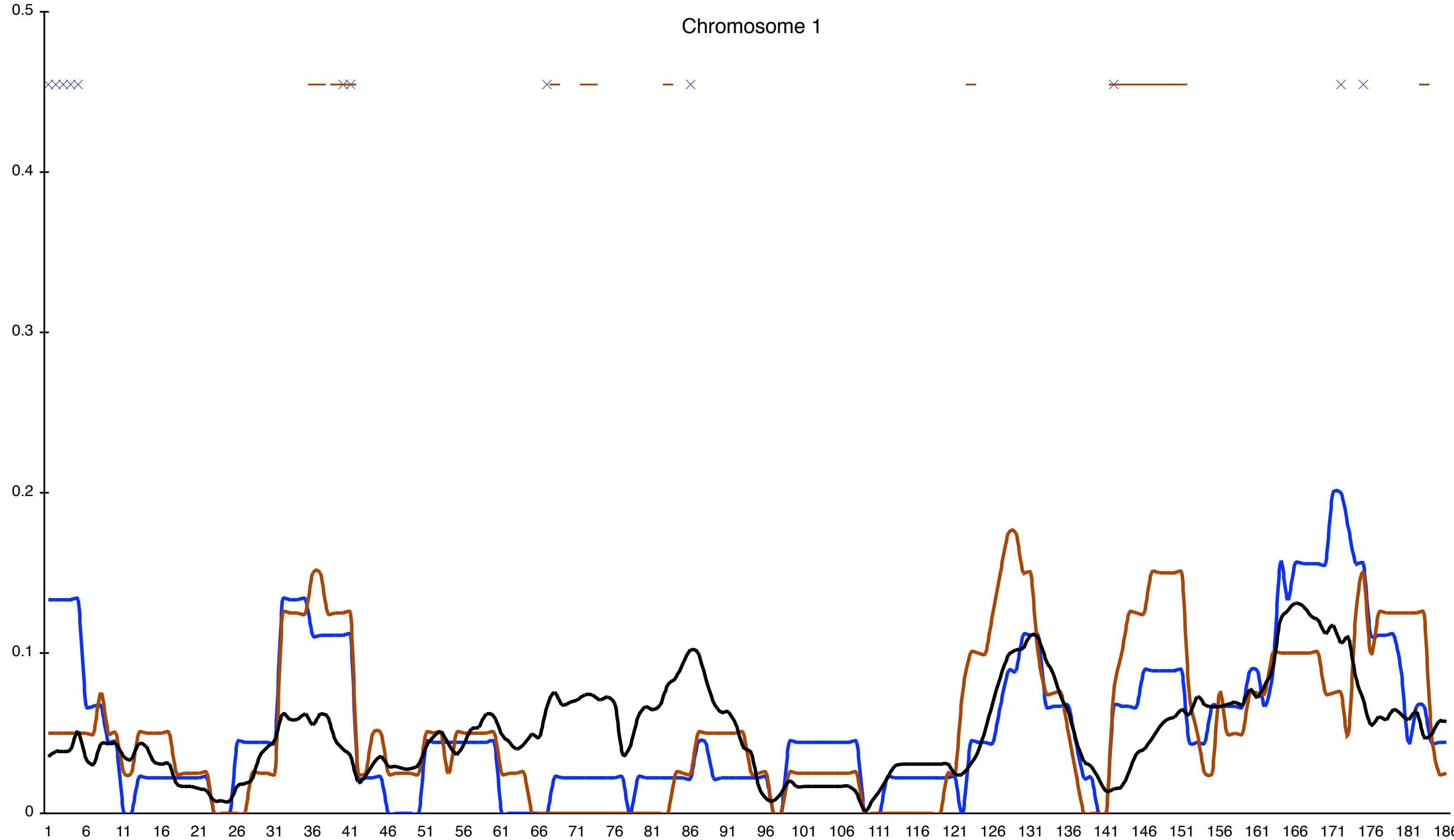
Chromosome 1
0.5
0.4
0.3
0.2
0.1
0
1
6
11
16
21
26
31
36
41
46
51
56
61
66
71
76
81
86
91
96
101
106
111
116
121
126
131
136
141
146
151
156
161
166
171
176
181
186

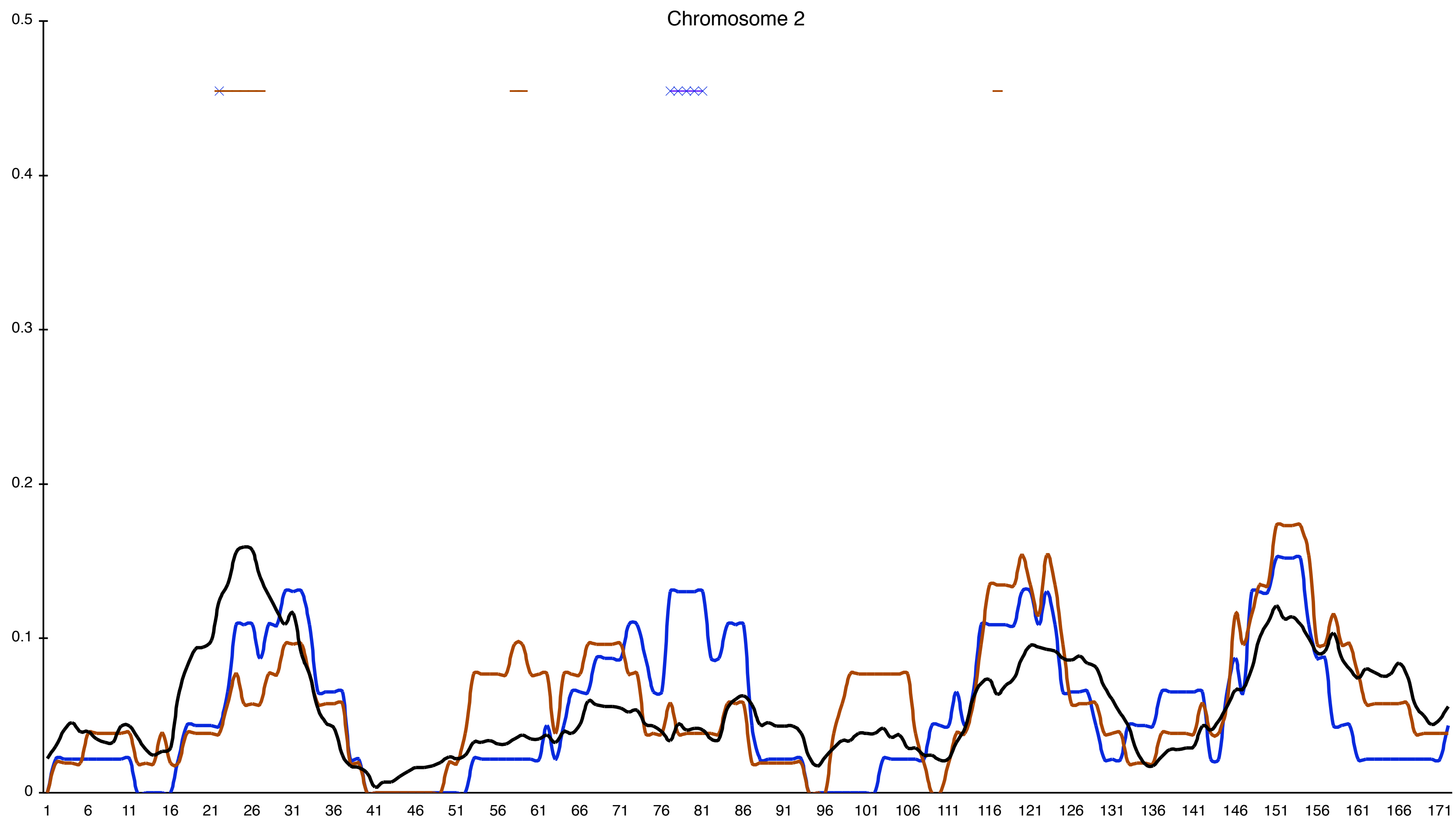

Chromosome 2
0.5
0.4
0.3
0.2
0.1
0
1 6 11 16 21 26 31 36 41 46 51 56 61 66 71 76 81 86 91 96 101 106 111 116 121 126 131 136 141 146 151 156 161 166 171

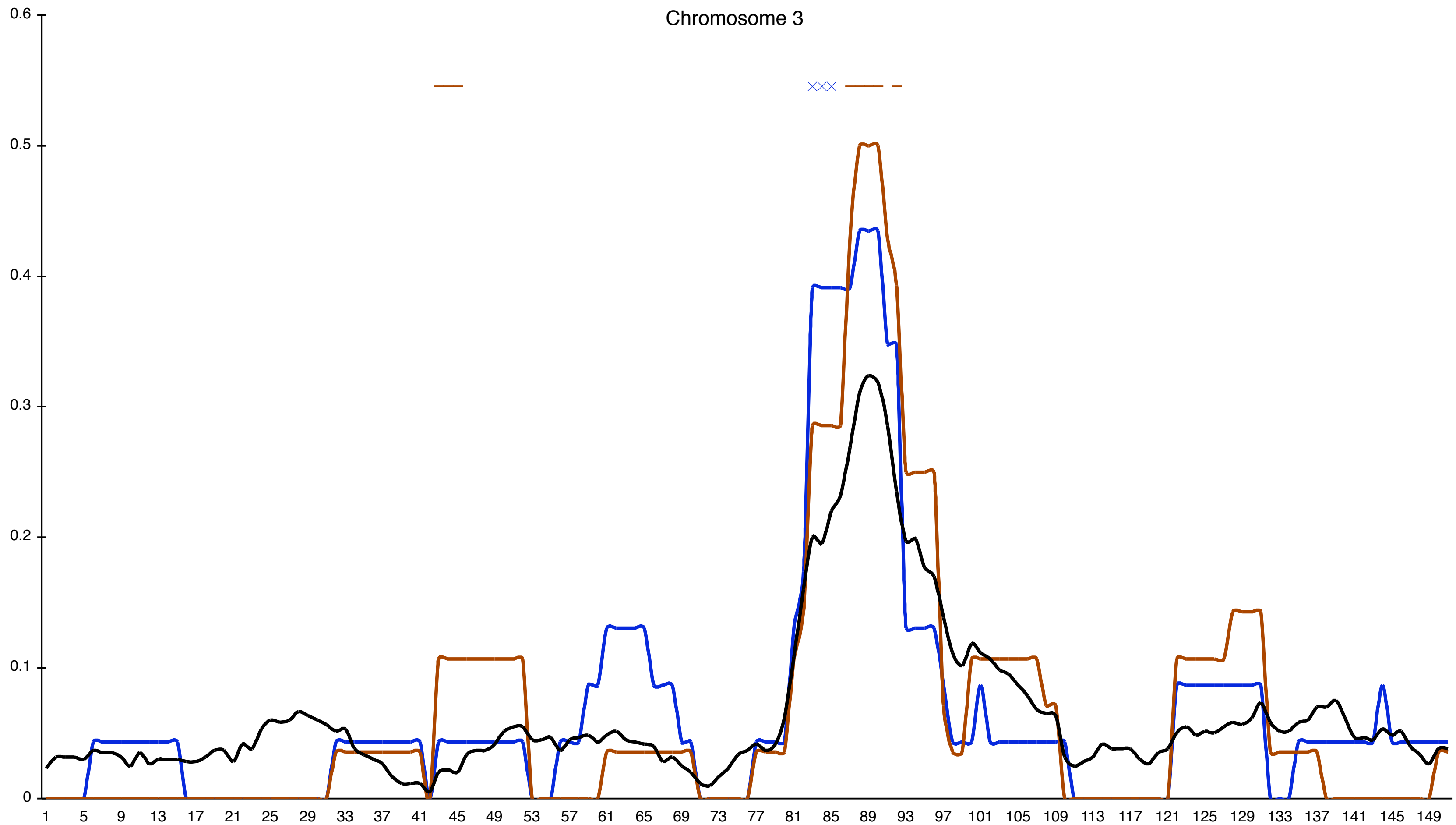

Chromosome 3
0.6
0.5
0.4
0.3
0.2
0.1
0
1
5
9
13
17
21
25
29
33
37
41
45
49
53
57
61
65
69
73
77
81
85
89
93
97
101
105
109
113
117
121
125
129
133
137
141
145
149

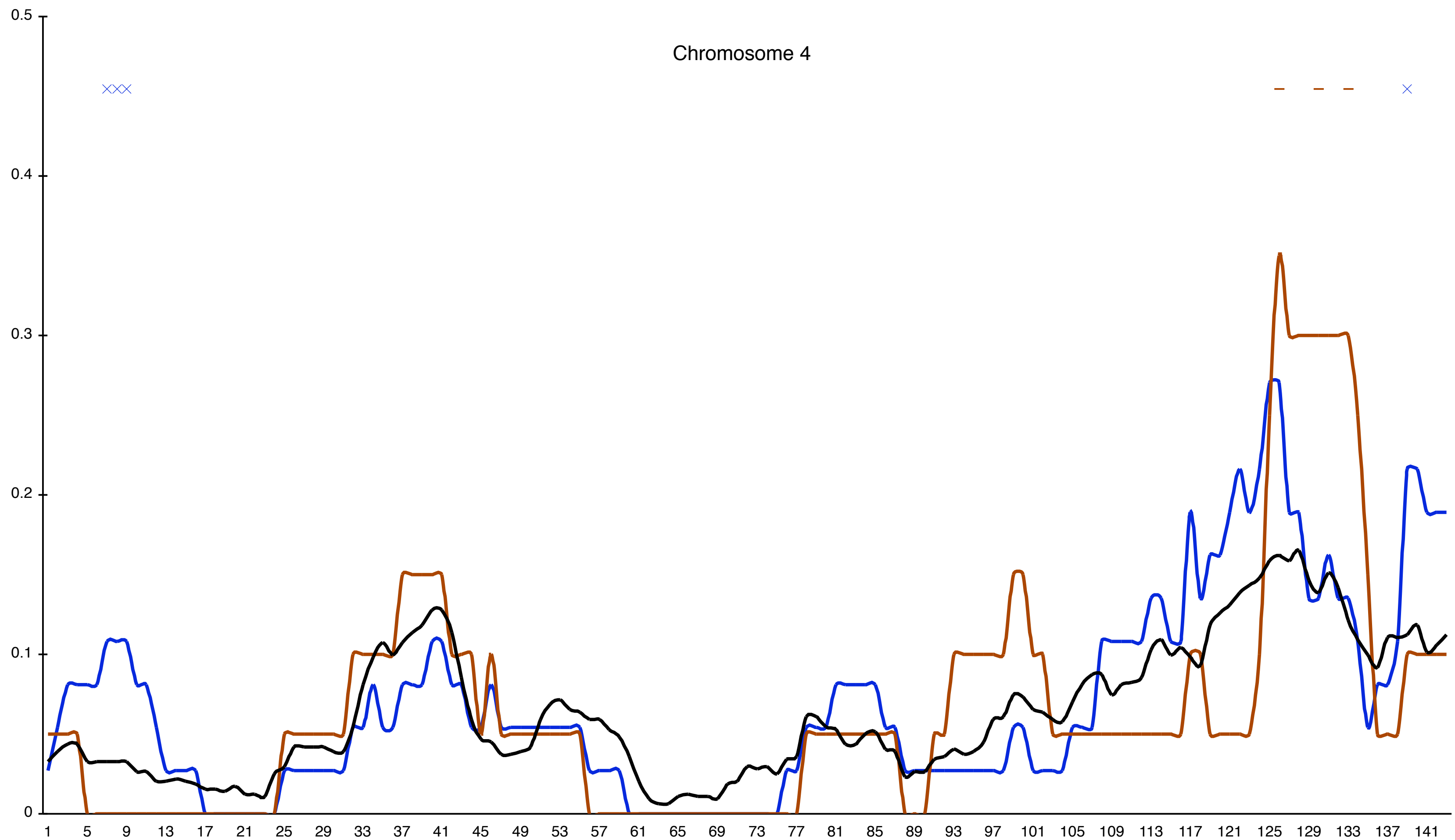

Chromosome 4
0.5
0.4
0.3
0.2
0.1
0
1
5
9
13
17
21
25
29
33
37
41
45
49
53
57
61
65
69
73
77
81
85
89
93
97
101
105
109
113
117
121
125
129
133
137
141

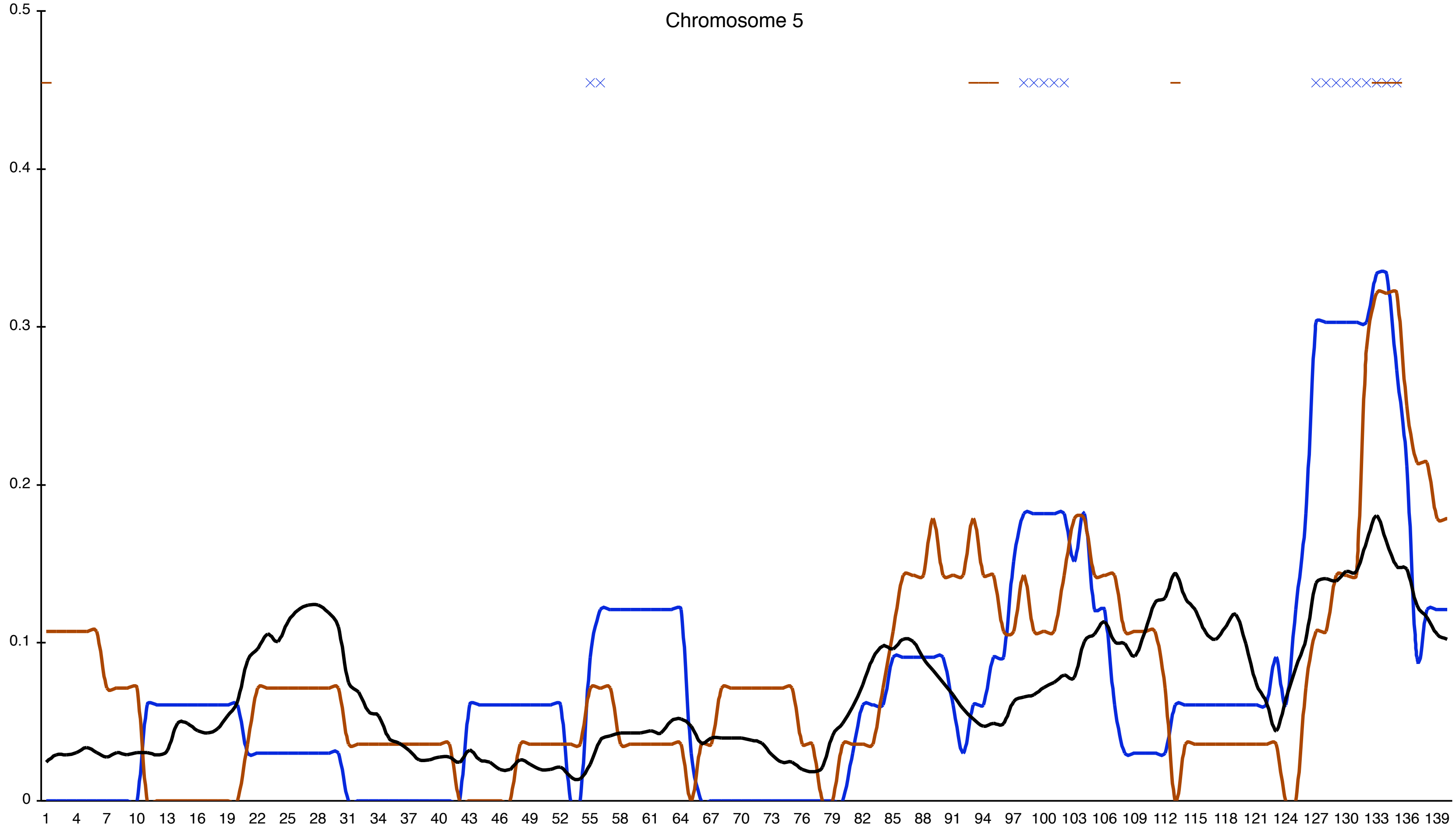
Chromosome 5
0.5
0.4
0.3
0.2
0.1
0
1 4 7 10 13 16 19 22 25 28 31 34 37 40 43 46 49 52 55 58 61 64 67 70 73 76 79 82 85 88 91 94 97 100 103 106 109 112 115 118 121 124 127 130 133 136 139

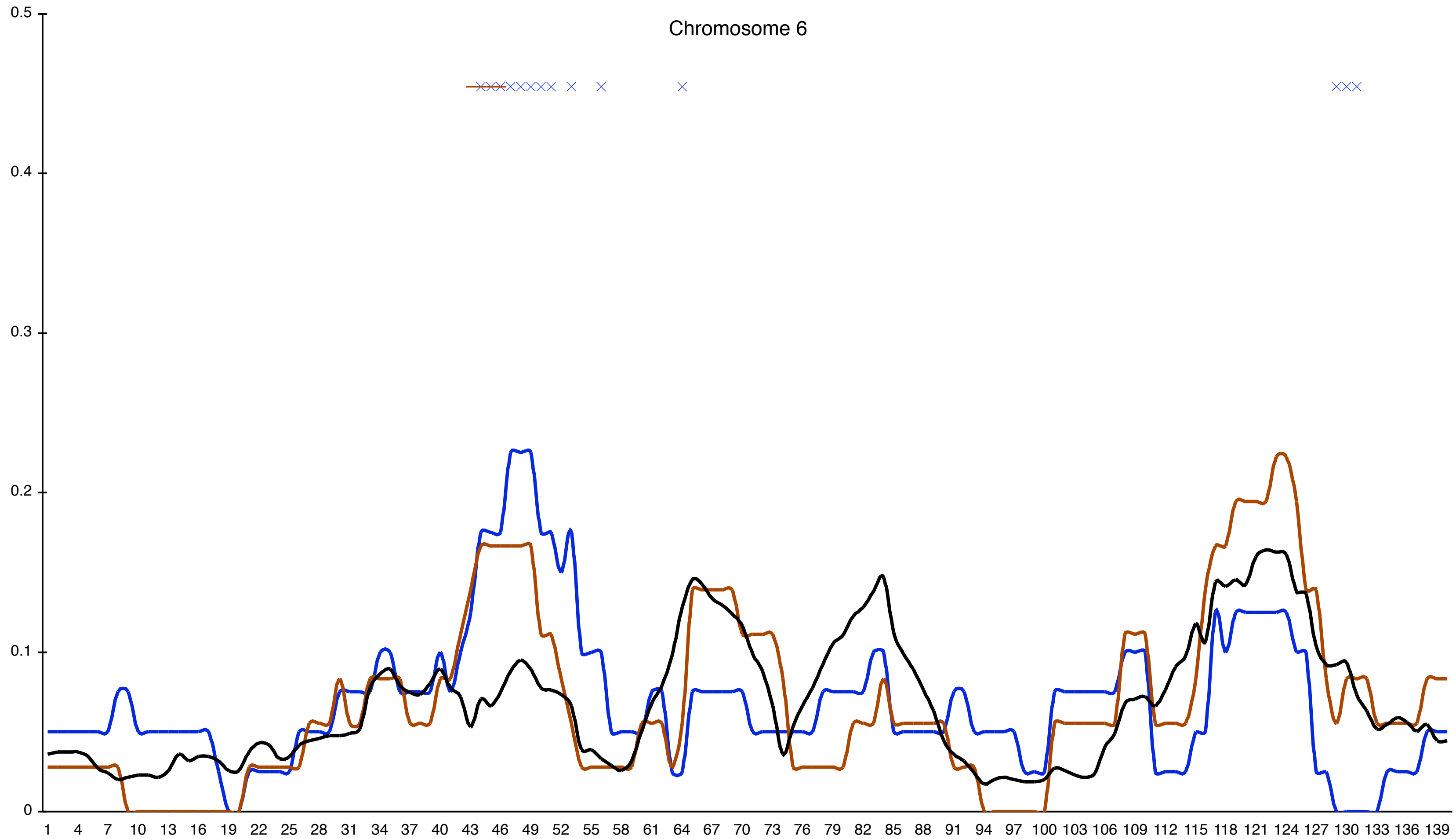
Chromosome 6
0.5
0.4
0.3
0.2
0.1
0
1 4 7 10 13 16 19 22 25 28 31 34 37 40 43 46 49 52 55 58 61 64 67 70 73 76 79 82 85 88 91 94 97 100 103 106 109 112 115 118 121 124 127 130 133 136 139

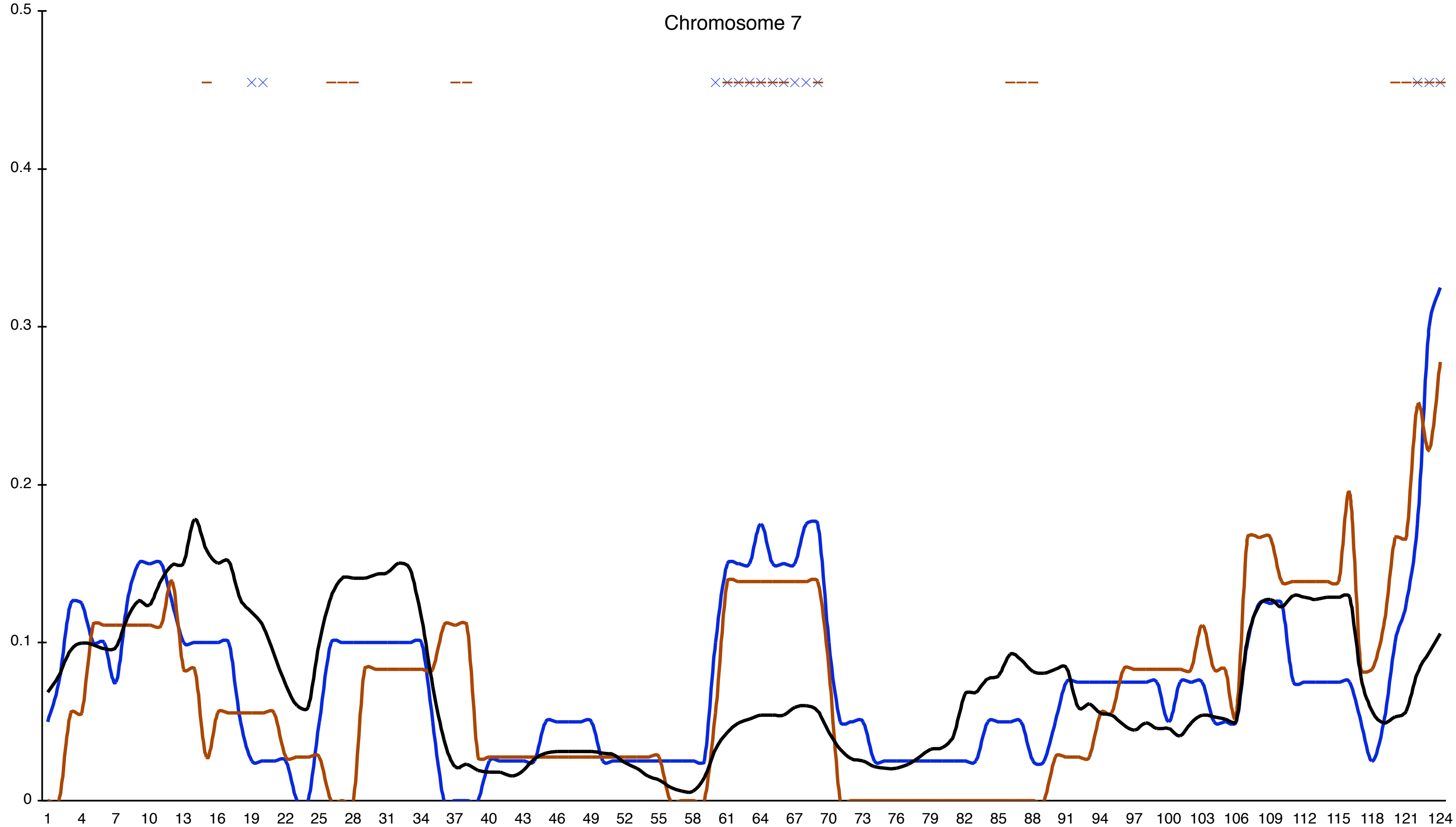

Chromosome 7
0.5
0.4
0.3
0.2
0.1
0
1 4 7 10 13 16 19 22 25 28 31 34 37 40 43 46 49 52 55 58 61 64 67 70 73 76 79 82 85 88 91 94 97 100 103 106 109 112 115 118 121 124

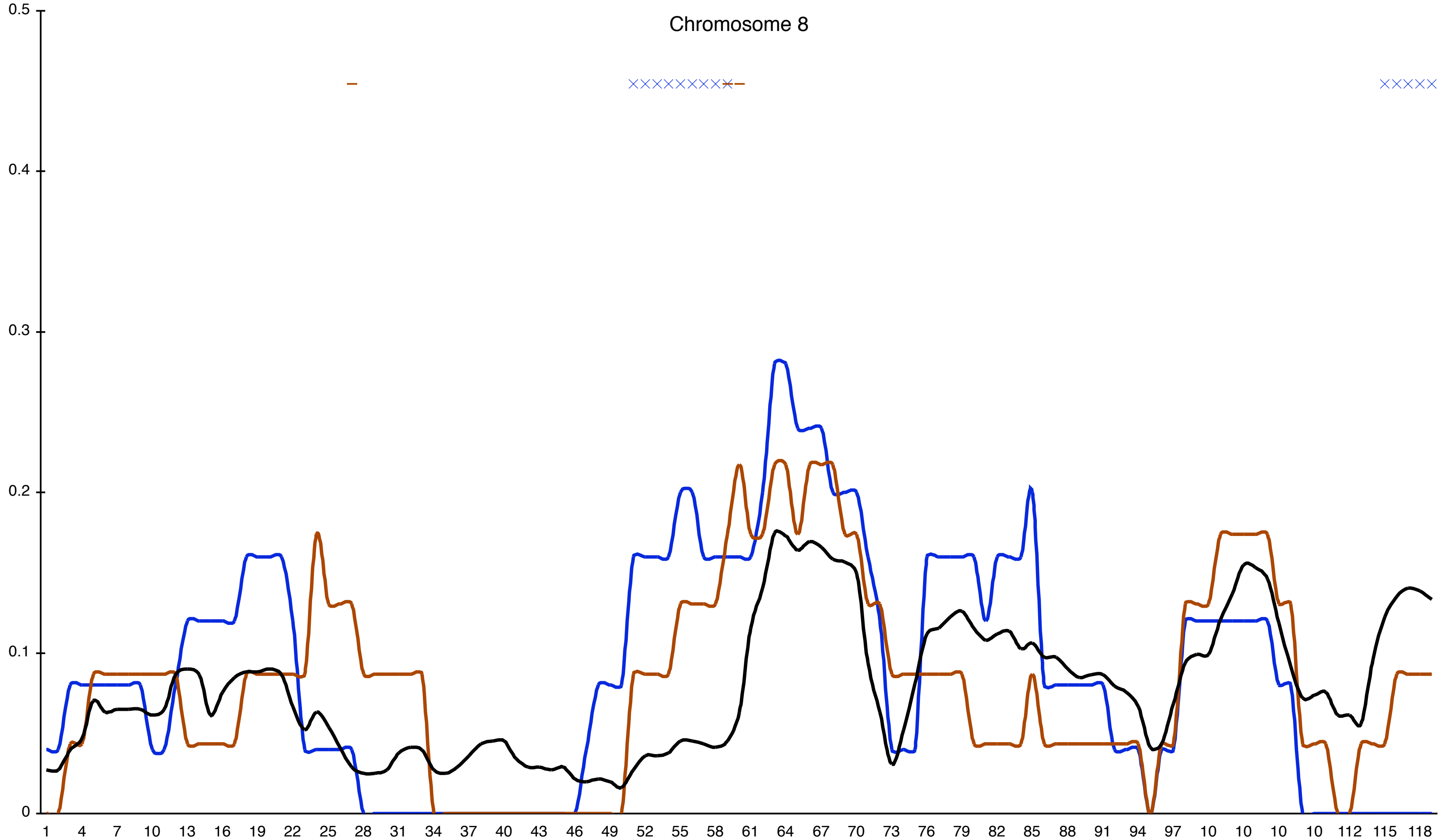

Chromosome 8
0.5
0.4
0.3
0.2
0.1
0
1 4 7 10 13 16 19 22 25 28 31 34 37 40 43 46 49 52 55 58 61 64 67 70 73 76 79 82 85 88 91 94 97 10 10 10 10 112 115 118

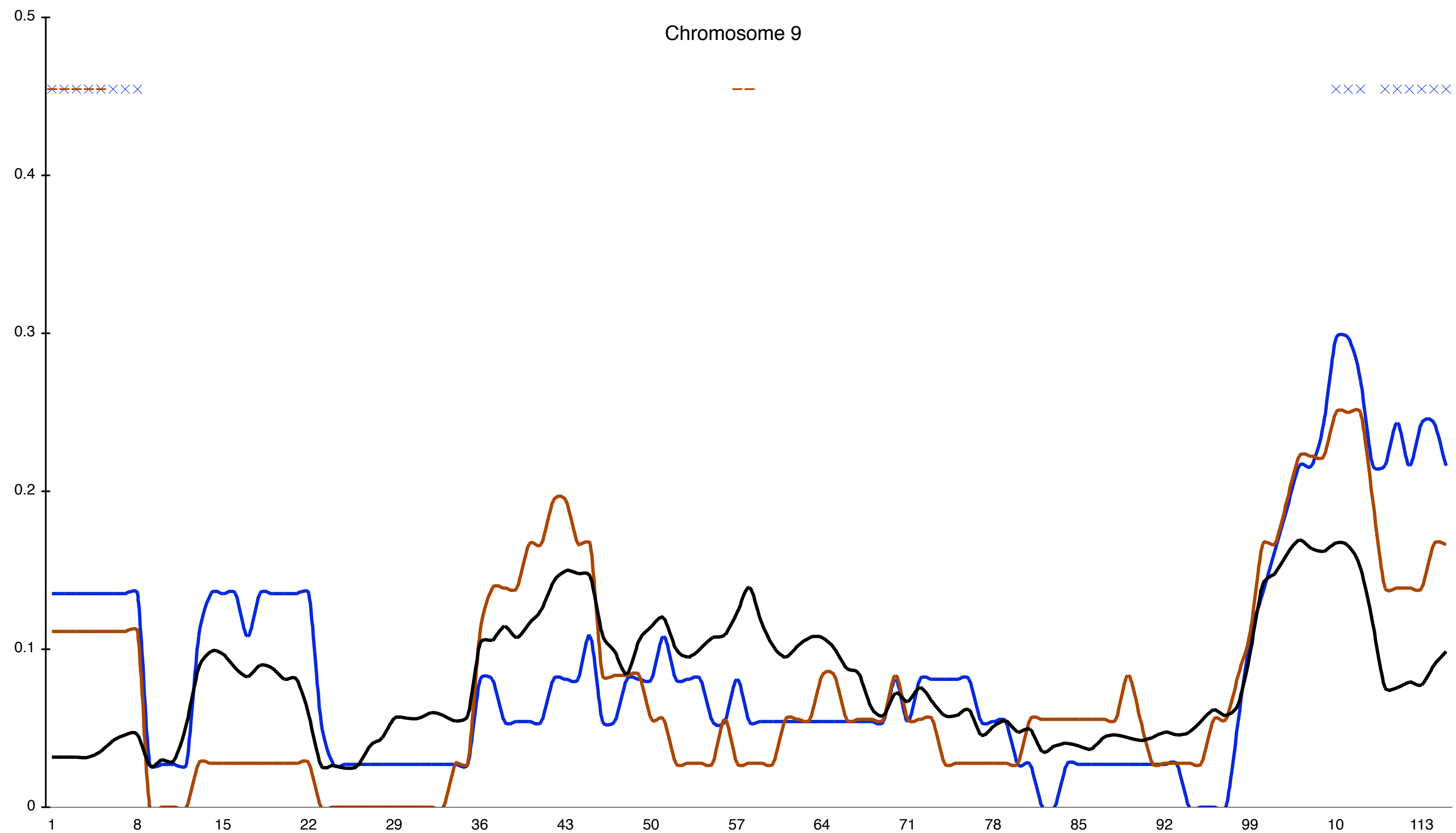
Chromosome 9
0.5
0.4
0.3
0.2
0.1
0
1
8
15
22
29
36
43
50
57
64
71
78
85
92
99
10
113

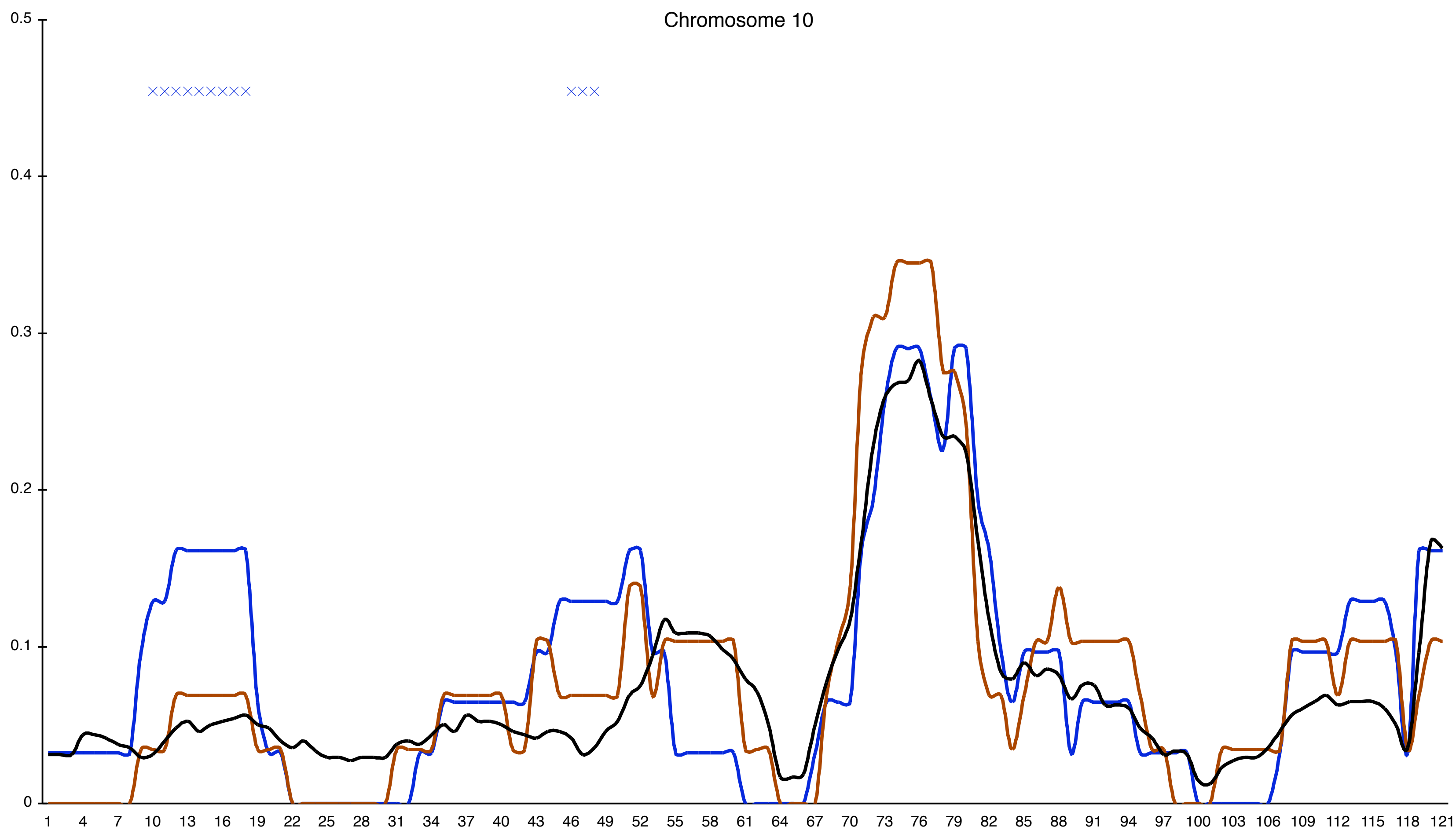

Chromosome 10
0.5
0.4
0.3
0.2
0.1
0
1 4 7 10 13 16 19 22 25 28 31 34 37 40 43 46 49 52 55 58 61 64 67 70 73 76 79 82 85 88 91 94 97 100 103 106 109 112 115 118 121

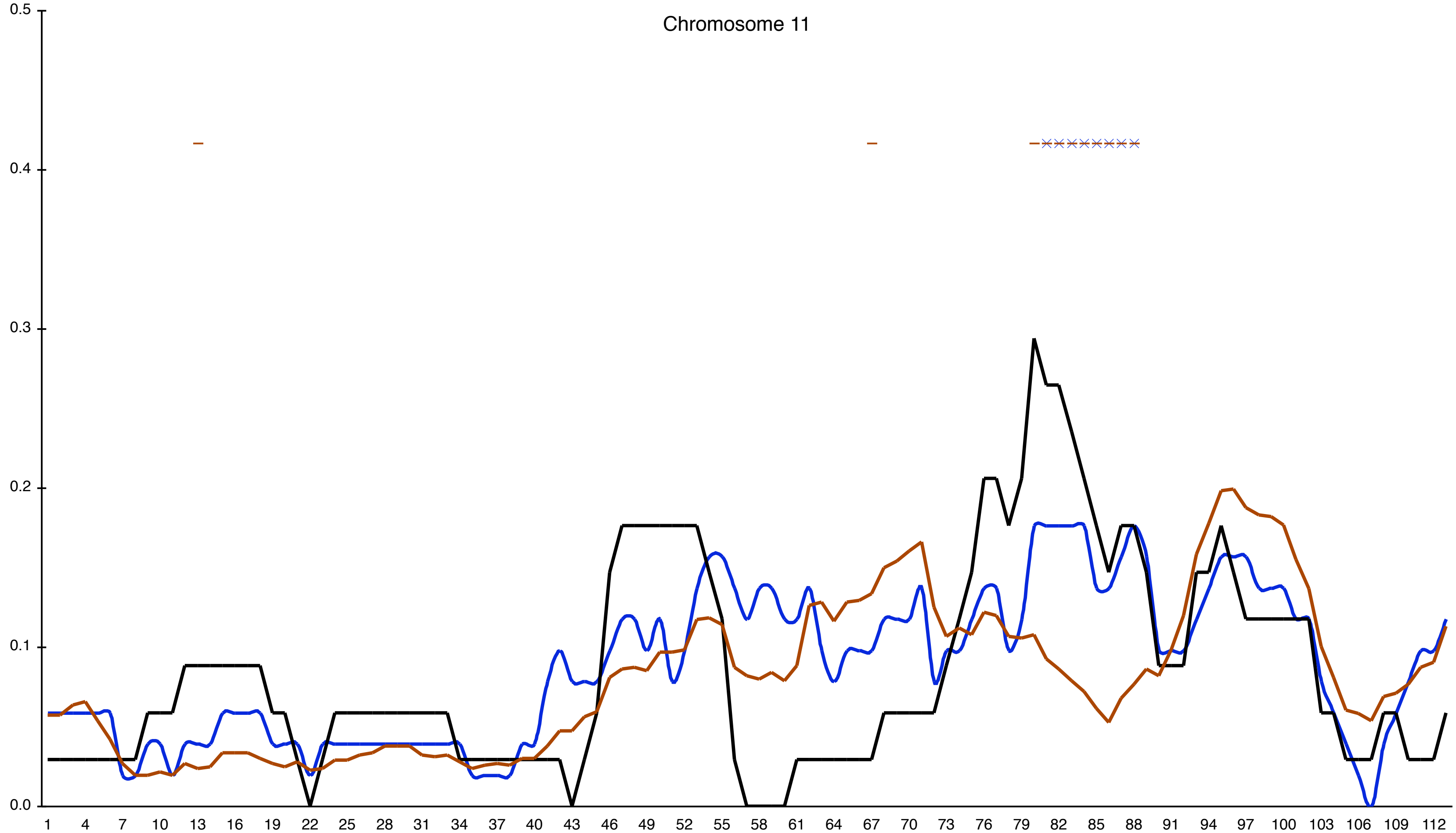

Chromosome 11
0.5
0.4
0.3
0.2
0.1
0.0
1 4 7 10 13 16 19 22 25 28 31 34 37 40 43 46 49 52 55 58 61 64 67 70 73 76 79 82 85 88 91 94 97 100 103 106 109 112

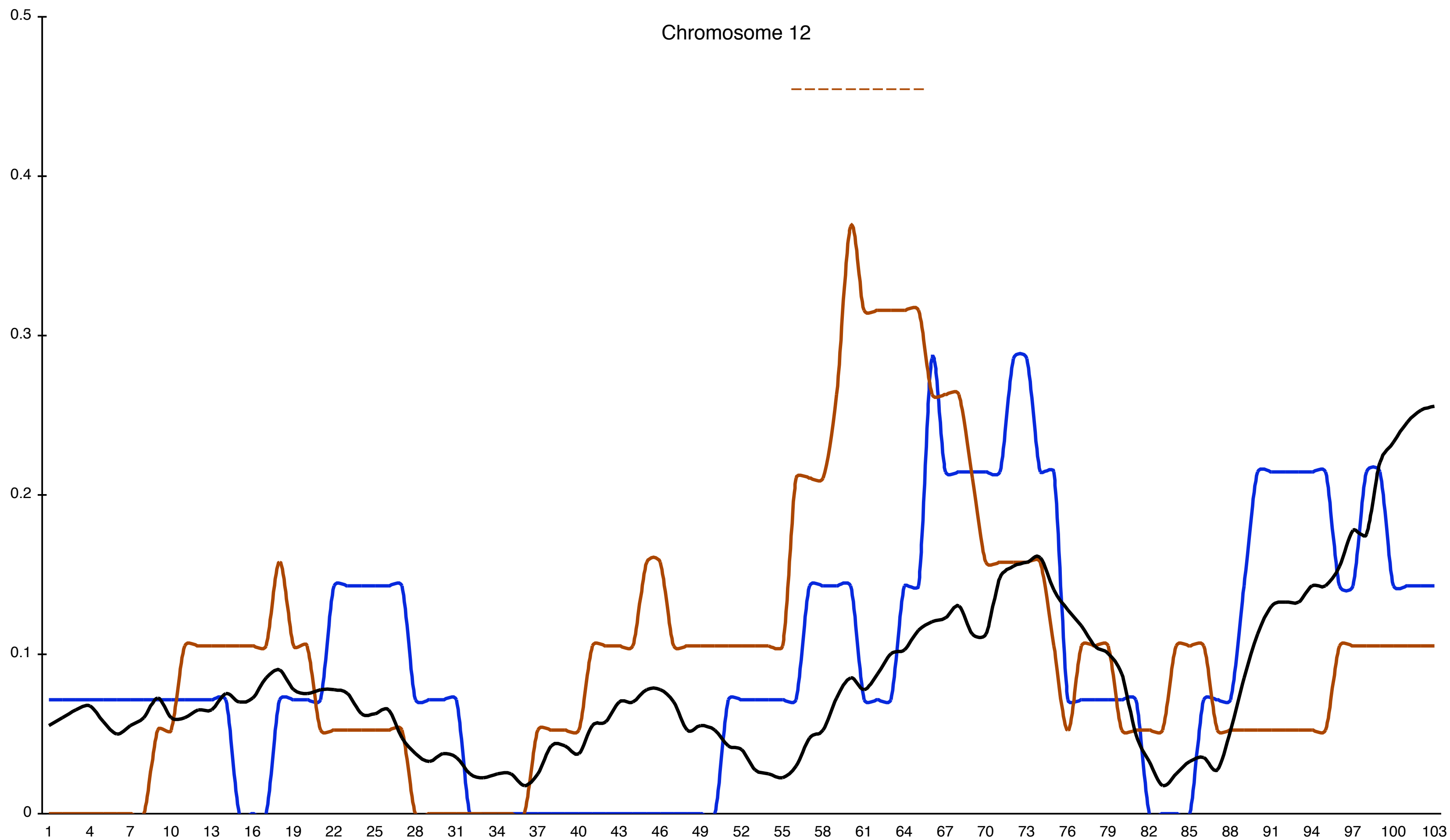
Chromosome 12
0.5
0.4
0.3
0.2
0.1
0
1 4 7 10 13 16 19 22 25 28 31 34 37 40 43 46 49 52 55 58 61 64 67 70 73 76 79 82 85 88 91 94 97 100 103

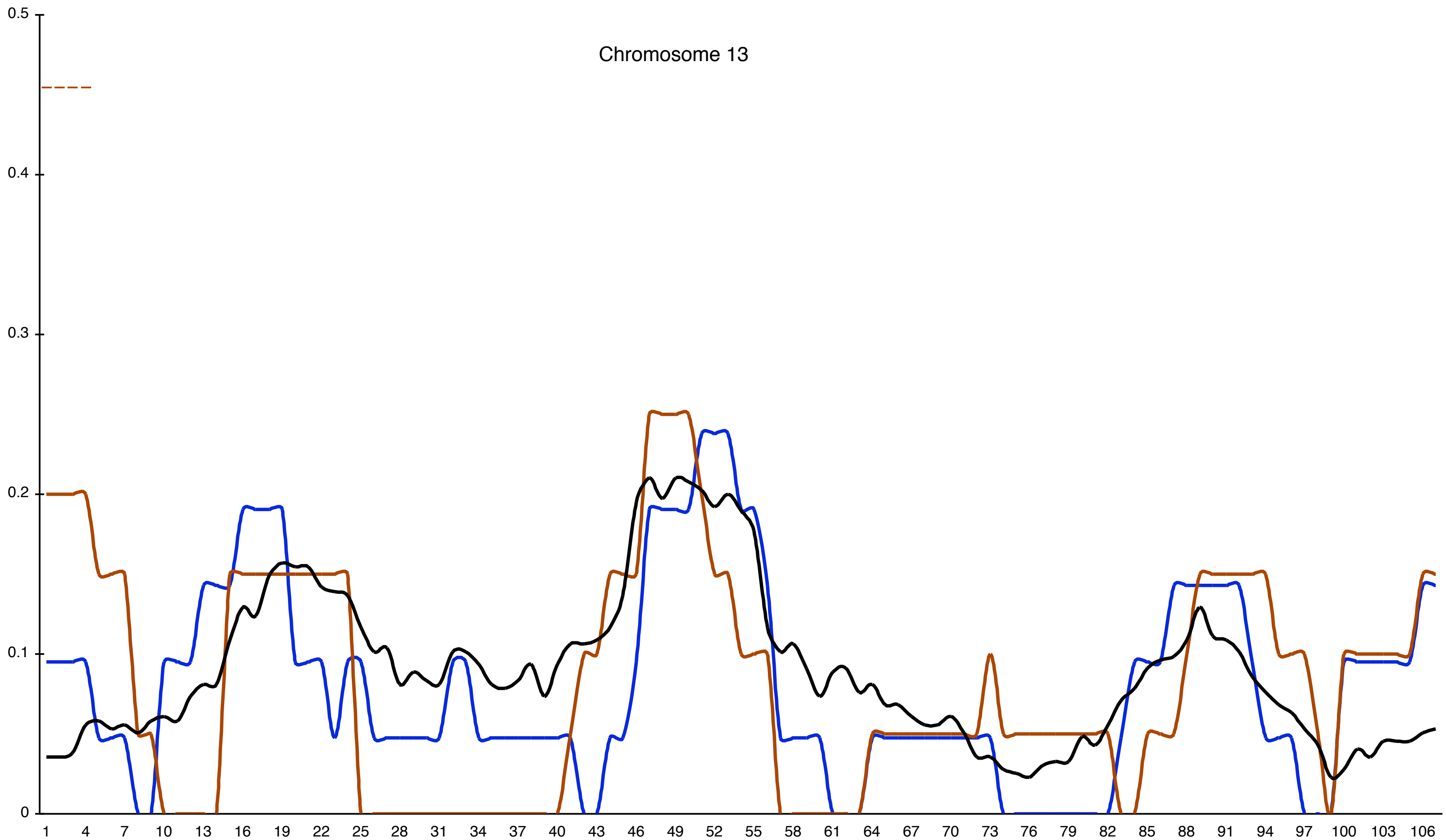

Chromosome 13
0.5
0.4
0.3
0.2
0.1
0
1
4
7
10
13
16
19
22
25
28
31
34
37
40
43
46
49
52
55
58
61
64
67
70
73
76
79
82
85
88
91
94
97
100
103
106

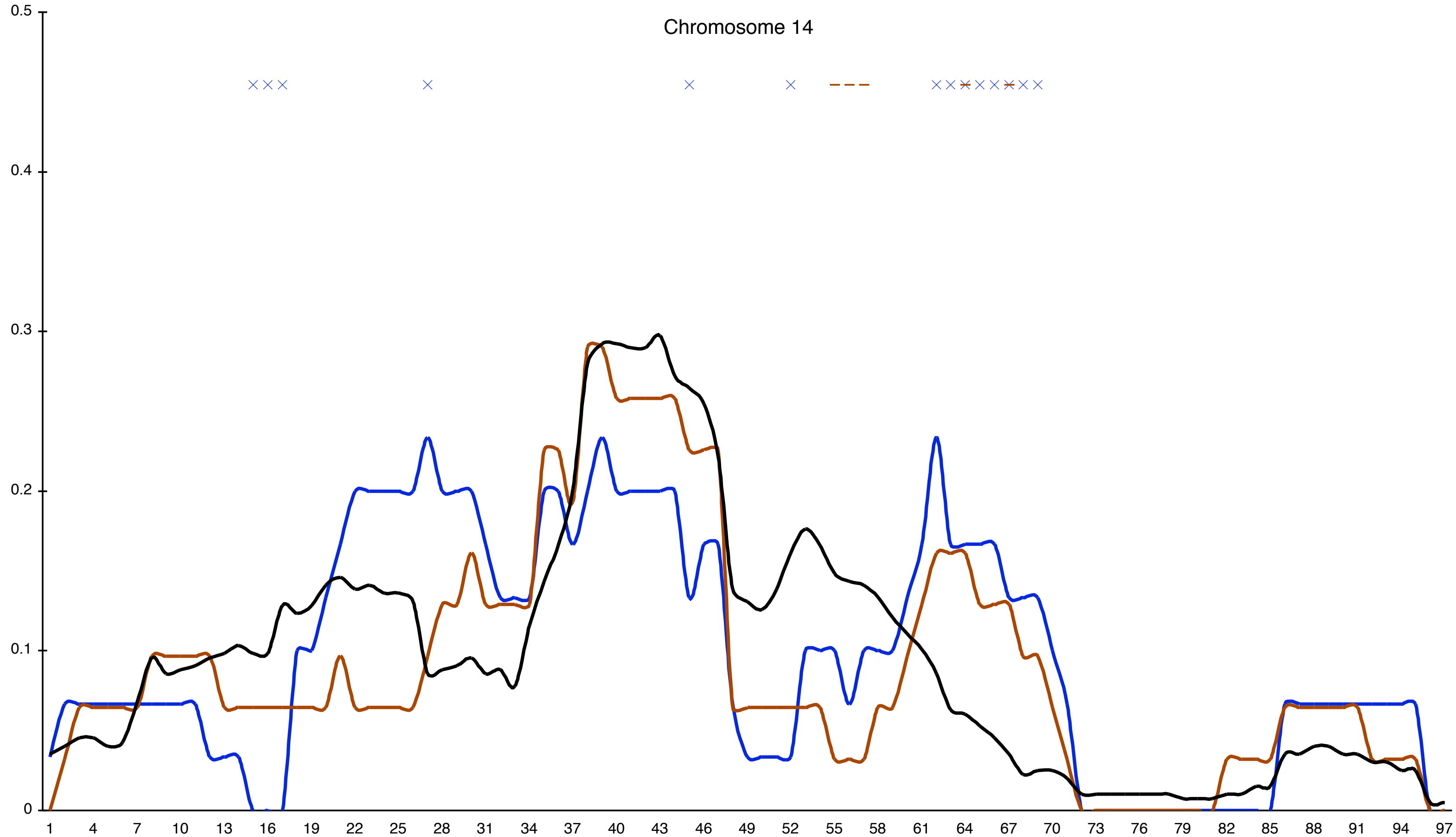

Chromosome 14
0.5
0.4
0.3
0.2
0.1
0
1
4
7
10
13
16
19
22
25
28
31
34
37
40
43
46
49
52
55
58
61
64
67
70
73
76
79
82
85
88
91
94
97

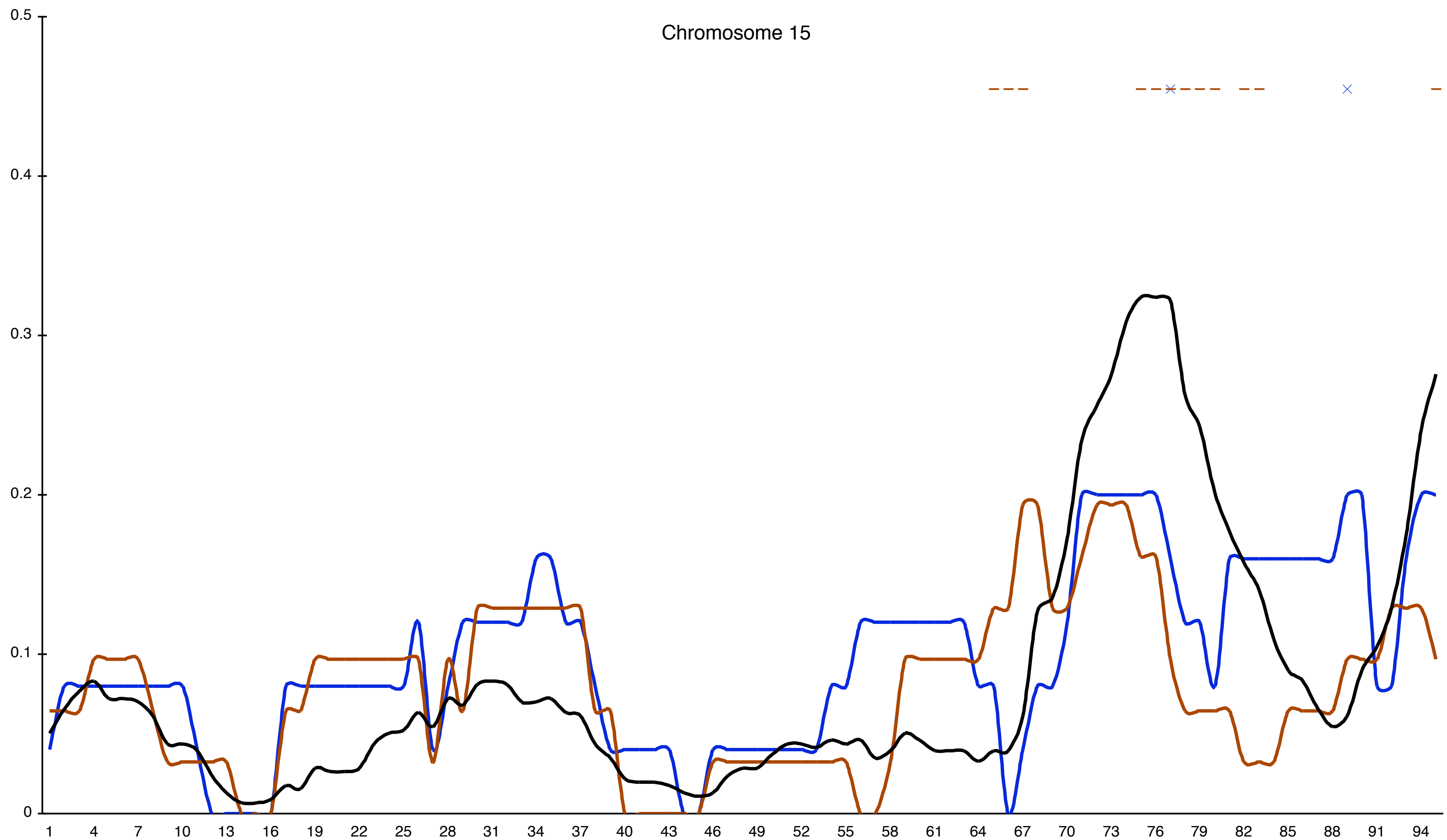
Chromosome 15
0.5
0.4
0.3
0.2
0.1
0
1 4 7 10 13 16 19 22 25 28 31 34 37 40 43 46 49 52 55 58 61 64 67 70 73 76 79 82 85 88 91 94

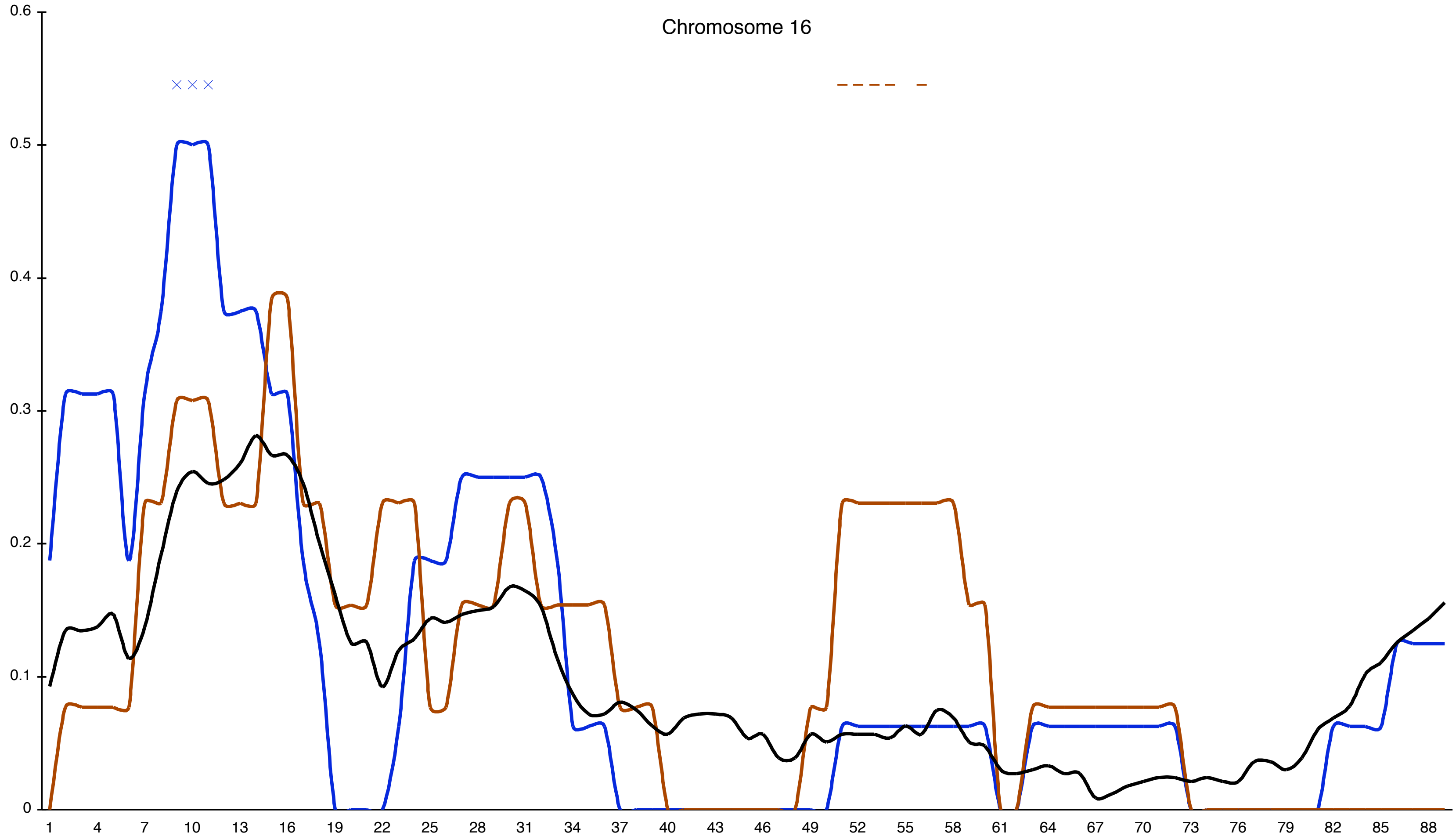

Chromosome 16
0.6
0.5
0.4
0.3
0.2
0.1
0
1
4
7
10
13
16
19
22
25
28
31
34
37
40
43
46
49
52
55
58
61
64
67
70
73
76
79
82
85
88

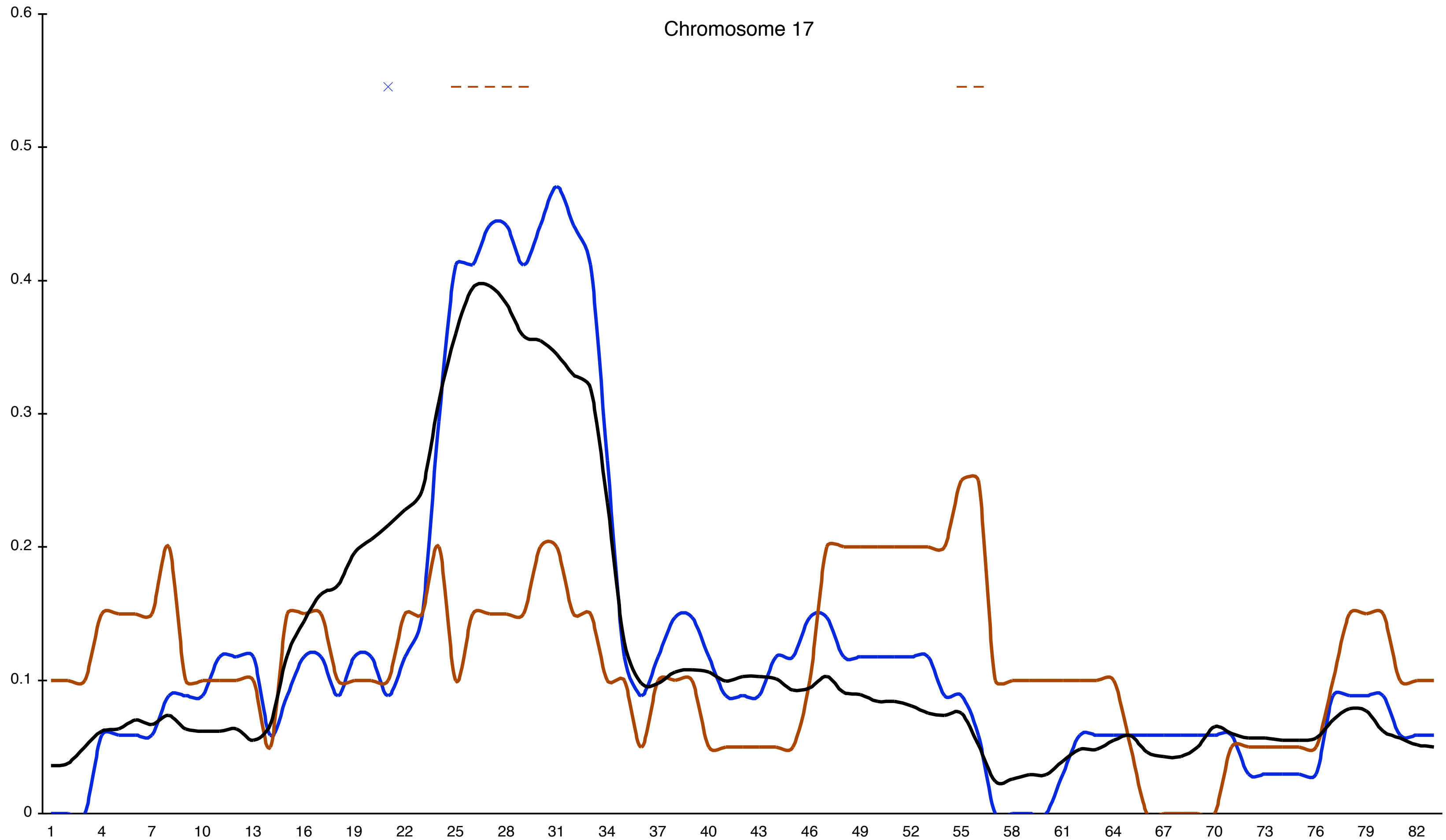

Chromosome 17
0.6
0.5
0.4
0.3
0.2
0.1
0
1 4 7 10 13 16 19 22 25 28 31 34 37 40 43 46 49 52 55 58 61 64 67 70 73 76 79 82

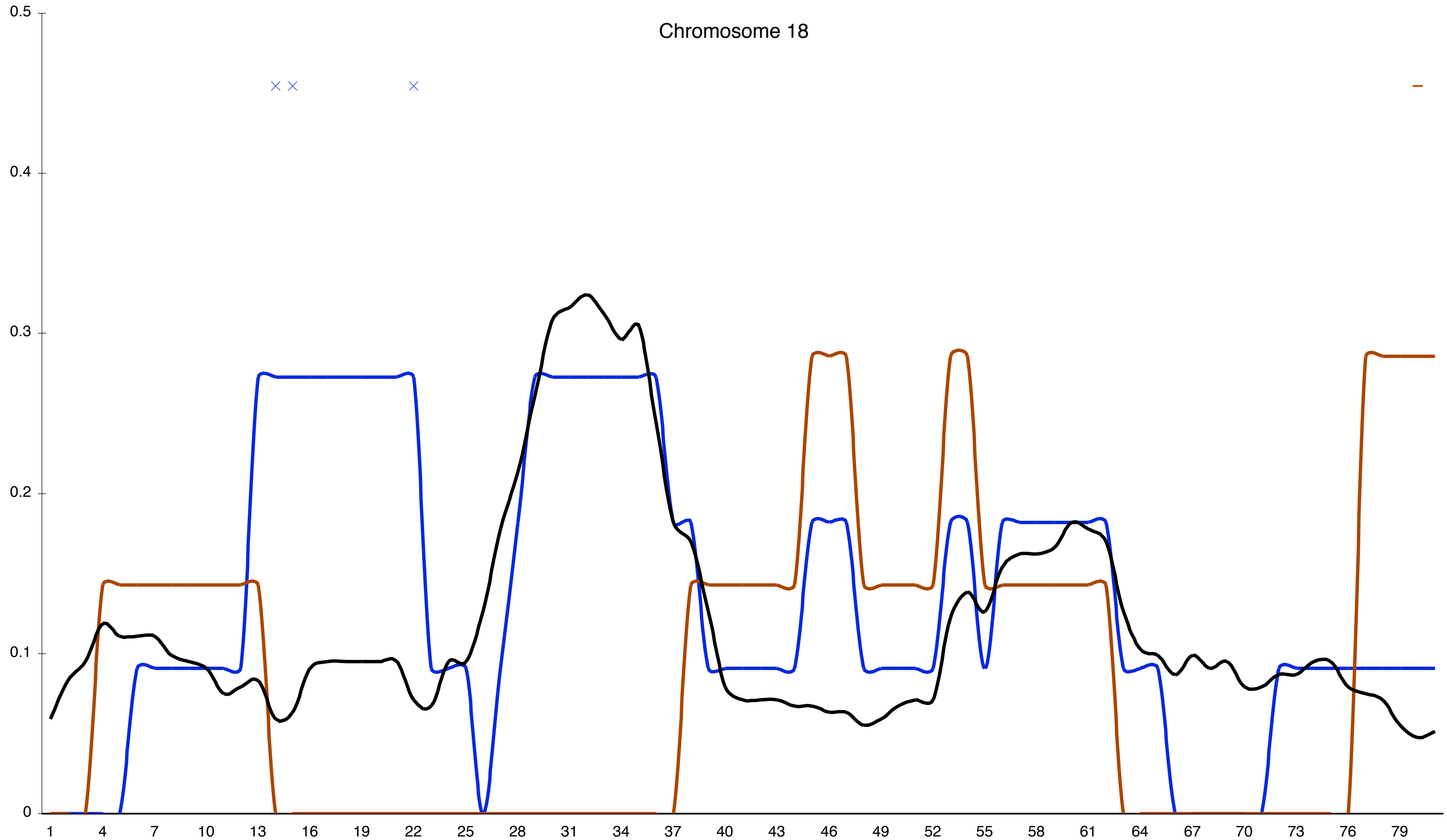

Chromosome 18
0.5
0.4
0.3
0.2
0.1
0
1
4
7
10
13
16
19
22
25
28
31
34
37
40
43
46
49
52
55
58
61
64
67
70
73
76
79

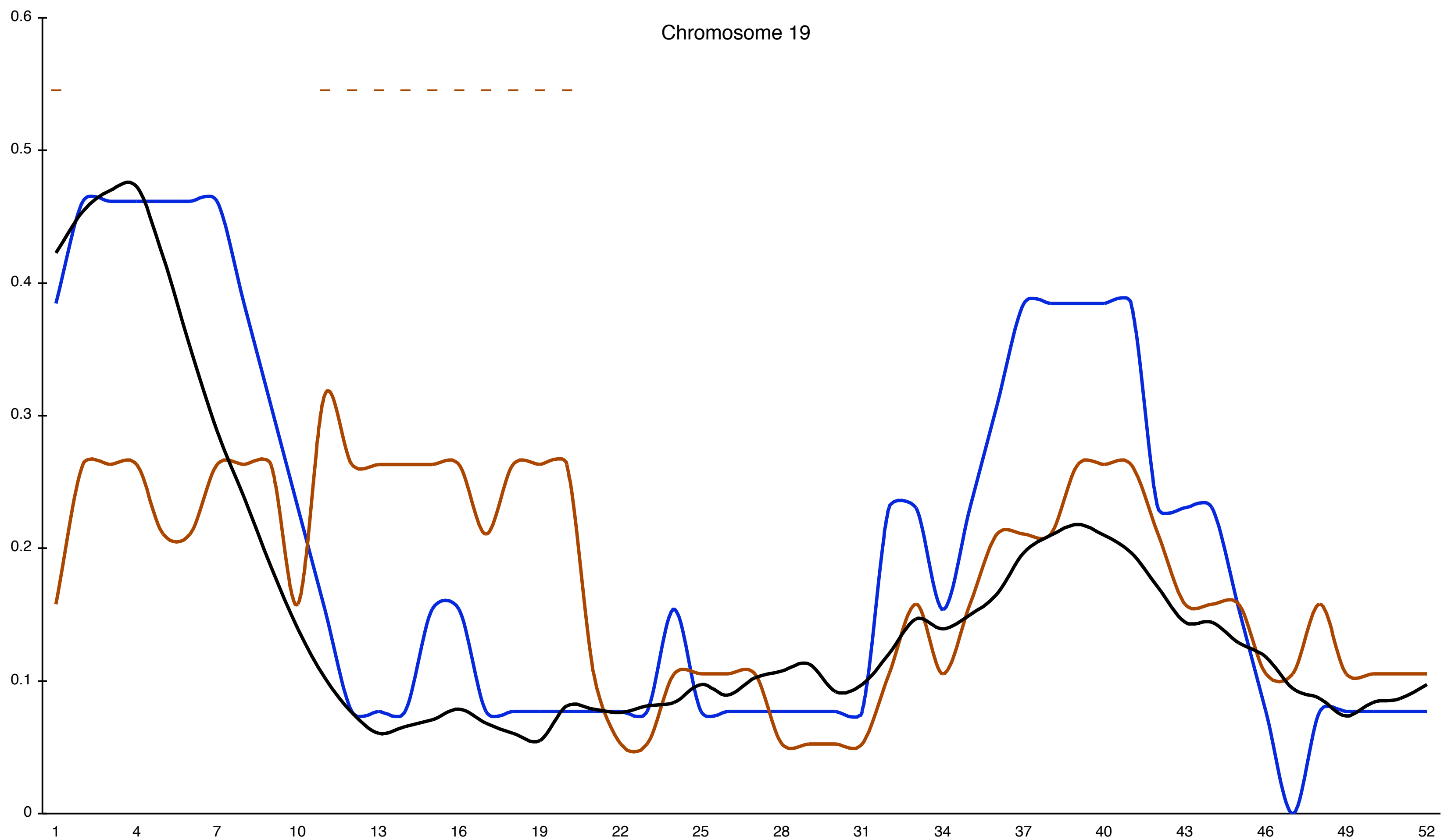

Chromosome 19
0.6
0.5
0.4
0.3
0.2
0.1
0
1
4
7
10
13
16
19
22
25
28
31
34
37
40
43
46
49
52

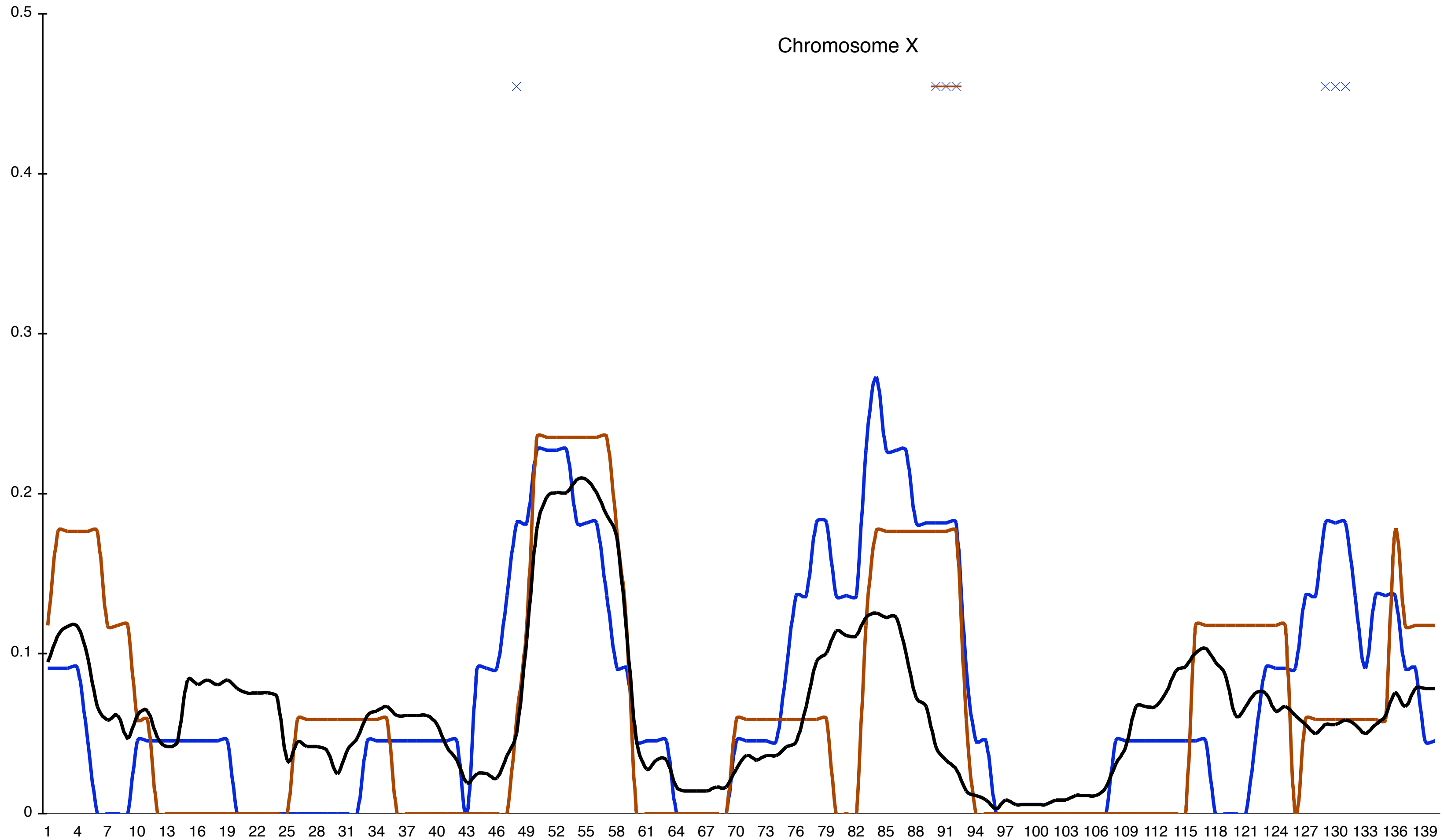
Chromosome X
0.5
0.4
0.3
0.2
0.1
0
1 4 7 10 13 16 19 22 25 28 31 34 37 40 43 46 49 52 55 58 61 64 67 70 73 76 79 82 85 88 91 94 97 100 103 106 109 112 115 118 121 124 127 130 133 136 139
