# Supplementary material for: Coordinate Gene Regulation during Hematopoiesis Is Related to Genomic Organization
Source: PLoS Biol. 2007 Nov 20;5(11):e309. doi: 10.1371/journal.pbio.0050309 (PMC2080650; doi:10.1371/journal.pbio.0050309)
Supplement: Supplementary file 2 [file pbio.0050309.sg009.pdf]

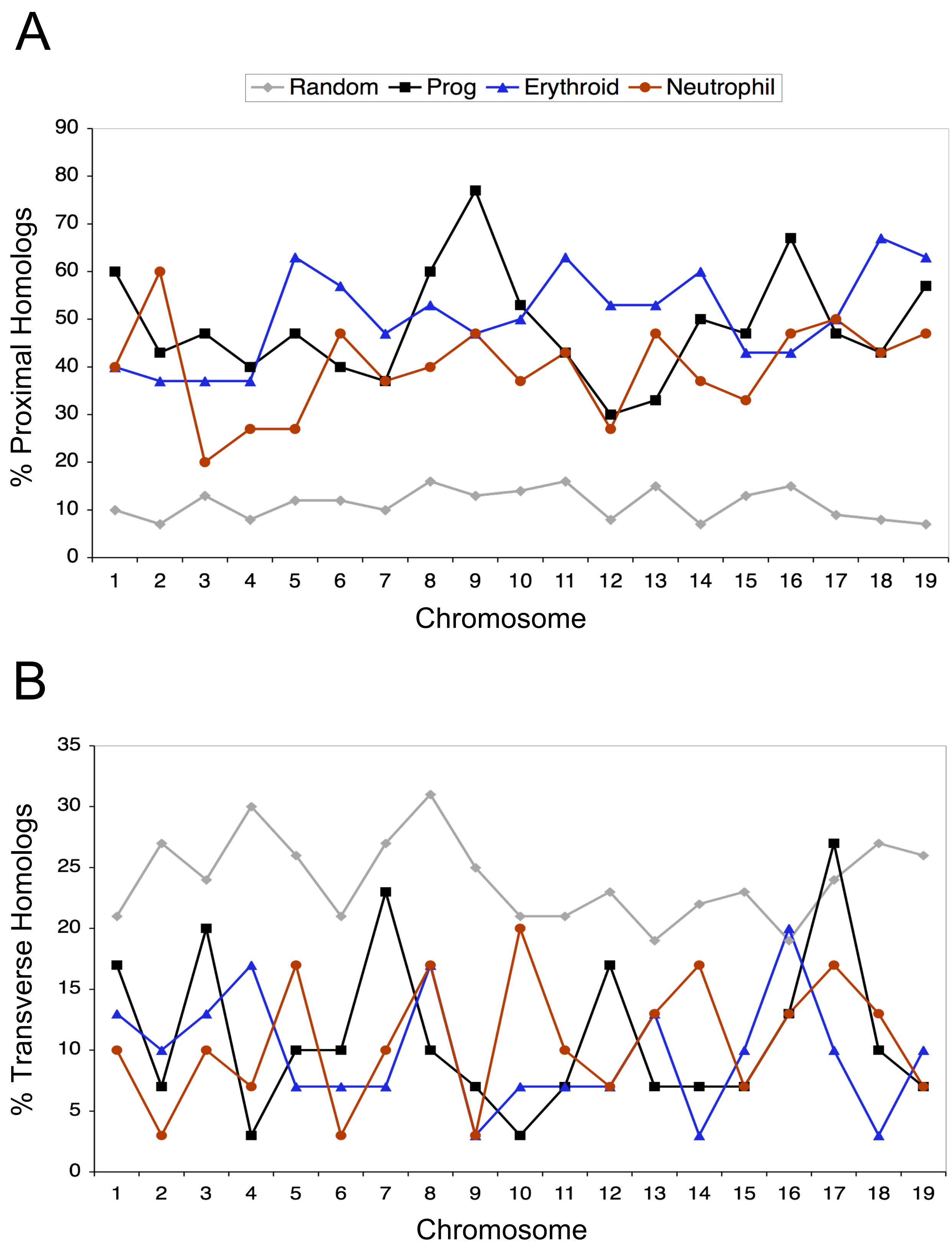

A
Random
Prog
Erythroid
Neutrophil
% Proximal Homologs
0
10
20
30
40
50
60
70
80
90
1
2
3
4
5
6
7
8
9
10
11
12
13
14
15
16
17
18
19
Chromosome
B
% Transverse Homologs
0
5
10
15
20
25
30
35
1
2
3
4
5
6
7
8
9
10
11
12
13
14
15
16
17
18
19
Chromosome
