# Supplementary material for: Coordinate Gene Regulation during Hematopoiesis Is Related to Genomic Organization
Source: PLoS Biol. 2007 Nov 20;5(11):e309. doi: 10.1371/journal.pbio.0050309 (PMC2080650; doi:10.1371/journal.pbio.0050309)
Supplement: Supplementary file 3 [file pbio.0050309.sd002.doc]

## Supplementary Information

***3-D FISH Analysis***

Probes were labelled by nick translation with biotin- and digoxigenin-conjugated nucleotides. Approximately 100ng of each probe were used per hybridization, along with 5µg murine Cot-1 DNA (Invitrogen) in 50% formamide/10% dextran sulfate/2x SSC. In all cases cells were allowed to adhere to slides for 4-5 minutes and then fixed for 10 minutes in 4% formaldehyde/PBS. Following washes in PBS cells were permeabilized in 0.5% saponin/0.5% Triton/PBS for 10 minutes, washed in PBS and equilibrated in 20%glycerol/PBS for >20 minutes. Slides were then frozen in liquid nitrogen and stored at –80˚C until use. Prior to hybridization slides were passed through three cycles of freeze-thawing in liquid nitrogen and then washed in PBS. Following 7 minutes in 0.1M HCl, the cells were subjected to a 1 hour RNase treatment (100μg/ml in 2xSSC) at 37˚C and washed in 2X SSC. DNA probes were denatured for 5 minutes >80˚C and pre-annealed for 20-30 minutes at 37˚C prior to application to slides. Cells were denatured for 3 minutes in 70% deionized formamide, 2X SSC, pH 7.0 at 75˚C, followed by 1 minute in 50% deionised formamide, 2X SSC, pH 7.0 at 75˚C. The probes were then added onto the slides with coverslips and allowed to hybridize overnight in a humid chamber at 37˚C. Coverslips were rinsed off in 2X SSC and the slides washed 3x in 50% formamide/2X SSC, pH 7 at 42˚C followed by two washes in 2X SSC. For probe detection, the slides were incubated for 25 minutes in blocking solution (5% milk in 4X SSC) followed by blocking solution containing Alexa-Fluor conjugated streptavidin and a monoclonal α-DIG antibody (Sigma). Slides were washed 3-4 times in 4X SSC/01%Triton X-100, counterstained with 4-,6-diamidino-2-phenylindole (DAPI, Sigma) and mounted in 90% glycerol, 0.1 M Tris–HCl, pH 8.0, and 2.3% DABCO (1,4-diazobicyclo-2,2,2-octane). Images were captured and analyzed as indicated in Materials and Methods.

## *Analysis of Chromosome Organization*

SVCell alpha prototype software (SVision LLC, Bellevue, WA) was used to assay spatial pattern associations between individual chromosomes across all the rosette images from the three lineages and a simulated rosette image set. SVCell is a next generation microscopy image informatics tool that contains fast image recognition algorithms, relational measurements, and supports the creation and review in real time of a large number of spatial patterns that can be derived from these relational measurements. SVCell image recognition and relational measurements automatically normalize the distortion and inter-sample variations among input images to make robust, accurate and comparable automated measurements across many samples. A spatial pattern of interest can be created (such as K nearest neighbor) and used to interrogate the experimental images to understand spatial pattern frequency in total and across all class interactions.

To characterize chromosome spatial pattern associations, two patterns were extracted for each chromosome class interaction (including same class homolog interactions) across the three lineage and simulated image sets. The steps in the pattern extraction process were: 1) detect rosette center, 2) find adjacency among individual chromosomes, 3) use the rosette center and individual chromosome adjacency information to generate relational measurements, object distance, and radial difference, 4) use the relational measurements to create two spatial patterns of proximal and transverse, and 5) create an SVCell recipe for batch execution of pattern scoring across the image sets.

SKY masks were created using the SKYView software (Applied Spectral Imaging) and exported as TIFs. As shown in Figure 3A, the SKY mask image contains the complete rosette where each pixel belonging to a chromosome territory is assigned an RGB value, and the background is black. Each chromosome class was assigned a unique RGB identifier. The SKY masks were imported to SVCell, which automatically recognized the chromosome mask region and class information, and stored the masks internally as a 24 bit mask image where each bitplane corresponds to a binary mask for a chromosome class (21 of the 24 planes were used).

The center of each rosette was determined automatically in SVCell. The procedure applies an outer distance transform to the chromosome masks. The outer distance transformation creates a distance image that assigns the shortest distance from each of the background pixels to a chromosome mask pixel. To remove image boundary regions from consideration, an initial threshold is applied to remove image regions of high distance. To find the rosette center, peaks in the distance image are detected. The centroid of the largest peak region was determined to be the rosette center. The center was labelled with an overlay, and a selection of study images were manually reviewed to confirm that center identification was performed correctly.

Each individual chromosome’s set of adjacent chromosomes was determined automatically in SVCell. The procedure uses a zone of influence (ZOI) based region partition, where the extent of the region partition is limited on the inside and outside of the rosette by two thresholds such that adjacency will not be calculated across the middle of the rosette or between chromosomes separated by a large background region (but not other chromosomes). The extent of the ZOI region is determined by applying the thresholds to the distance image produced in the ZOI operation.

The ZOI operation creates an unambiguous representation across which adjacency transitions can be calculated. If at least one pixel of two chromosomes’ partitioned regions touch, then they are considered adjacent. To illustrate, Figure 6A shows an input SKY mask image, and Figure 6B shows the same mask image after the ZOI operation. Touching regions in 6B are considered adjacent. ZOI images for a selection of study images were reviewed manually to confirm both that the ZOI operation is a reasonable method to determine adjacency, and that the adjacency calculation was performed correctly. Next the relational measurements object distance and radial difference were calculated using the rosette center and the adjacency information. Object distance is defined as the number of chromosomes between two chromosomes of interest (adjacent chromosomes’ object distance equals one). For each of the 40 individual chromosomes in the input SKY mask image, its object distance to each of the other 39 individual chromosomes was calculated. Object distance of any two individual chromosomes is calculated as the shortest path through the ZOI image.

The radial difference relational measurement is calculated as the smallest angle in radians between any two chromosomes measured from the centroid of each chromosome mask through the center of the rosette. For each of the 40 chromosomes, its 39 radial difference relational measurements are calculated between it and all of the other chromosomes in the image. SVCell was then used to derive two spatial patterns from these relational measurements, and then automatically score the occurrence of the spatial pattern in the image sets.

To determine a spatial pattern of proximal, a spatial pattern rule was created in SVCell using the object distance relational measure. For each chromosome, its set of proximal chromosomes was defined as those chromosomes whose object distance was less than 4 (note that there are multiple chromosomes at each object distance 1 through 3). Each proximal occurrence for a class interaction was counted as a single occurrence. The number of proximal occurrences was tallied for all chromosome class interactions, including same class homolog interactions. The proximal spatial pattern rule, method for homolog data integration and image set data accumulation rule were saved as an SVCell recipe. The recipe was then applied to each of the four image sets consisting of at least thirty rosettes each for fully automated execution of adjacency pattern scoring in batch mode. Results are tallied across the image sets and presented in Figure 6C and Figure S9A. Recipes measuring direct proximity and once-removed were also established and utilized (Dataset S1).

To determine a pattern of transverse, a spatial pattern rule was created in SVCell using the radial difference relational measure. For each chromosome, its set of transverse chromosomes was defined as those chromosomes whose radial difference measure was greater than 0.83333p radians. Because the radial difference measure is always calculated as the smallest angle between the two chromosomes, this pattern rule creates a window of +/-.16667p directly across the rosette from the chromosome of interest. Each transverse occurrence for a class interaction was counted as a single occurrence. The number of transverse occurrences was tallied for all chromosome class interactions, including same class homolog interactions. An SVCell recipe was created and then applied to all four image sets for fully automated execution of the transverse pattern scoring in batch mode. Results are tallied across the image sets and presented in Figure 6C and Figure S9B.

For the analysis of total gene expression and CT localization, the TIF masks were used as inputs to a script running in SVCell that divided the nuclear mask into three concentric regions of equivalent area, and determined the number of pixels from each chromosome territory mask that were in each of the three nuclear regions. The three concentric nuclear regions of equal area were determined by an inner distance transform operation performed on the input nuclear mask. The inner distance transformation creates a distance image that assigns the shortest distance from each of the mask object pixels to the nuclear boundary. The concentric nuclear region assignment is based on the pixel’s distance value. The assignment function is determined by performing a histogram operation on the distance image pixels to assign them to three equal size populations in order of distance value. The fractional part of cut-off distances required the allocation of some pixels of equal distance to different nuclear regions. This was accomplished by evenly eroding the ring of pixels at the cut-off distance by the amount required for a perfect distribution, given an under-segmenting adjustment of 0 to 2 pixels (the total object area modulo 3). Lastly, the number of pixels in each expression area or CT mask that overlapped with each nuclear region were counted and reported, yielding three numbers per CT mask, which is summarized in Figures 3, 4B, and S4. The amount of CT in each region for each chromosome in each lineage was then analyzed by ANOVA with Bonferroni post-test (Dataset S1).
